# A thermostable Cas9-based genome editing system for thermophilic acetogenic bacterium *Thermoanaerobacter kivui*

**DOI:** 10.1128/aem.01170-25

**Published:** 2025-09-08

**Authors:** Yilin Le, Xue Liu, Shidong Zhou, Pengju Wu, Mengqi Zhang, Jianzhong Sun, Jinfeng Ni, Huilei Wang

**Affiliations:** 1 Biofuels Institute, School of Emergency Management, School of the Environment and Safety Engineering, Jiangsu University 506427, Zhenjiang, Jiangsu, PR China; 2 State Key Laboratory of Microbial Technology, Shandong University 214177 , Qingdao, Shandong, PR China; Kyoto University, Kyoto, Japan

**Keywords:** thermophilic acetogens, *Thermoanaerobacter kivui*, thermophile, thermostable Cas9, genome editing

## Abstract

*Thermoanaerobacter kivui* is a thermophilic acetogenic bacterium capable of thriving at elevated temperatures up to 66°C. It metabolizes carbohydrates such as glucose, mannose, and fructose and can also grow lithotrophically utilizing hydrogen (H_2_) and carbon dioxide (CO_2_) or carbon monoxide (CO), with acetate serving as its main product. A simple and efficient genome editing system for *T. kivui* would not only facilitate the understanding of the physiological function of enzymes involved in energy and carbon metabolism but also enable metabolic engineering. To address this issue, we developed a thermostable Cas9-based genome editing system for targeted gene knockout and gene integration into the *T. kivui* genome. Gene knockout assays were conducted on the *adh* gene, responsible for encoding alcohol dehydrogenase, and the *ldh* gene, encoding lactate dehydrogenase. Furthermore, the *adhE* gene from *Thermoanaerobacter ethanolicus*, which encodes a bifunctional aldehyde/alcohol dehydrogenase enzyme, was successfully integrated into the *T. kivui* genome. As a result, the engineered strain was able to produce ethanol. Following a liquid culturing period with kanamycin sulfate for about 72 hours, the efficiency of gene editing was enhanced, resulting in a ratio of mutants out of all colonies obtained of 90%. The results confirm the validity and efficiency of the thermostable Cas9-based genome editing system in *T. kivui* for gene editing.

**IMPORTANCE:**

Thermophilic acetogenic microorganisms represent an emerging metabolic engineering platform for the production of various biochemicals from hydrogen and carbon dioxide, or synthesis gas, under conditions of high-temperature fermentation. Gas fermentation has gained significant research interest due to its excellent thermodynamics, economic feasibility, and multisubstrate utilization. However, a major obstacle to the use of thermophilic acetogenic microorganisms as metabolic engineering platforms is the scarcity of genetic tools. This study demonstrates a proof of concept for a thermostable Cas9-based genome editing of the thermophilic acetogenic bacterium *T. kivui*. The system is an important expansion to the genetic toolbox of *T. kivui*, enabling a better understanding of key enzyme functions and the construction of cell factories for the biotechnological conversion of carbon dioxide and organic substrates into value-added products.

## INTRODUCTION

*Thermoanaerobacter kivui*, a thermophilic acetogenic bacterium, is capable of heterotrophic or autotrophic growth on a mineral medium without the need for additional vitamin supplements (1). The optimal temperature for growth of *T. kivui* is 66°C, with a pH optimum of 6.4. Organic substrates that support growth include glucose, mannose, fructose, pyruvate, and formate, with acetate being the primary product (1–3). It has a faster doubling time of about 2 hours on hydrogen (H_2_) plus carbon dioxide (CO_2_) compared to other thermophilic acetogens (1). Due to these advantages, *T. kivui* is a promising candidate for producing industrial chemicals at high temperatures (4, 5).

However, unlike other acetogens (such as *Clostridium ljungdahlii* [6] and *Moorella thermoacetica* [7]), *T. kivui* lacks the potential to produce ethanol due to the absence of aldehyde dehydrogenase or aldehyde:ferredoxin oxidoreductase (8). Acetate is the primary end product of *T. kivui*. Therefore, it is crucial to use genetic engineering technology to modify its metabolic pathway and shift its end products from acetate to a more valuable chemical. On the other hand, the physiological functions of certain crucial enzymes related to the Wood–Ljungdahl pathway, energy metabolism, and the Embden–Meyerhof–Parnas pathway remain unclear. While several key enzymes have been purified and characterized *in vitro* in *T. kivui* (9, 10), physiological functions of some key enzymes remain poorly understood (10–15). The functions of certain enzymes have been elucidated through the generation of markerless mutants utilizing the gene encoding orotate phosphoribosyltransferase (*pyrE*) as a selective marker (16).

Genetic manipulation of thermophiles remains a technical challenge (17, 18). Factors that may influence the development of genetic manipulation of a thermophile include marker selection, DNA uptake ability, and vector stability (19, 20). Several genetic systems have been developed for the genetic engineering of mesophilic acetogens (21) and methanogenic archaea (22). In particular, CRISPR/Cas-based genome editing technologies have been employed in mesophilic acetogens for the purpose of modifying the metabolic pathway (23–25).

Compared to the genetic systems available for mesophilic acetogens, those for thermophilic acetogens are still relatively scarce. In 2018, Basen et al. developed a markerless gene deletion and integration system for *T. kivui* based on nutritional selection (16). The basic genetic manipulation technology developed by the Muller and Basen groups is suitable for studying the physiological function of a number of enzymes, including 1-phosphofructosekinase (16), mannitol-1-phosphate dehydrogenase (2), hydrogen-dependent carbon dioxide reductase (HDCR) (26), monofunctional CO dehydrogenase (Coos) (27), and energy-converting hydrogenase (Ech2) (28).

However, CRISPR/Cas9-based genome editing systems have not been developed in thermophilic acetogens. The development of a CRISPR/Cas-based genetic system would represent an important expansion to the genetic tool box of the thermophilic acetogenic bacterium *T. kivui*. Cas9 from *Streptococcus pyogenes* has not been used for genome editing in an obligate thermophile because it is not active at an elevated temperature. Fortunately, several thermostable Cas9 proteins have been characterized (29, 30). CRISPR/Cas-based genetic systems have been successfully applied for genome editing in various thermophilic bacteria, including *Bacillus smithii* ET 13831 (29), *Clostridium thermocellum* (31), *Thermus thermophilus* (32), *Thermoanaerobacter ethanolicus* (33), and *Thermoanaerobacterium aotearoense* (34). However, editing efficiency varies among different thermophiles due to differences in DNA uptake capacity and homologous recombination (HR) performance.

This study aimed to establish a thermostable Cas9-based genome editing system in *T. kivui*. The system’s efficacy was successfully demonstrated through carefully designed gene knockout and knock-in experiments.

## RESULTS AND DISCUSSION

### Uptake of plasmids containing the thermostable Cas9 gene into *T. kivui*

Heterologous expression of Cas9 in host cells is critical for establishing a Cas9-based genome editing system. A previous report indicated that the CRISPR/Cas9 system for *Clostridium autoethanogenum* exhibited poor efficiency, which was likely due to uncontrolled expression of Cas9 (25). In our previous study, the shuttle expression vector, designated pBlu10-Htk, was registered in public databases (GenBank: MN843970). A plasmid, designated pBlu10-Slay-Cas9 (33), has been constructed based on the shuttle expression vector pBlu10-Htk. The plasmid pBlu10-Slay-Cas9 harbors a thermostable Cas9 gene driven by the S-layer promoter (Pslay), a thermostable gram-positive origin of replication derived from plasmid pMU131, and a thermostable kanamycin resistance marker. The plasmid pBlu10-Slay-Cas9 was successfully introduced into *T. ethanolicus* JW200 cells via natural competence-mediated transformation (33).

In this experiment, *T. kivui* cells were transformed by natural transformation with the plasmid pBlu10-Slay-Cas9 containing a thermostable Cas9 gene under the control of a native constitutive promoter (Pslay) from *T. ethanolicus* JW200. The presence of the plasmid in the kanamycin-resistant isolates was verified by re-isolation of the plasmid followed by PCR using primers K1 and K2. The results demonstrated that *T. kivui* cells were capable of taking up the plasmid pBlu10-Slay-Cas9, although the transformation efficiency was relatively low, at approximately 200 CFU/μg of DNA. In comparison, the transformation efficiency of a control vector, pBlu10-Htk, which lacks Cas9 and gRNA, was approximately 1,200 CFU/μg of DNA. In addition, a vector pBlu10-S-P-gH with Cas9 and gRNA without homology arms was transformed into *T. kivui* cells. The transformation efficiency decreased further to about 50 CFU/μg of DNA.

*T. kivui* is a bacterial species belonging to the genus *Thermoanaerobacter*. However, it exhibits significant differences in physiological characteristics compared to other *Thermoanaerobacter* spp. (8). Previous reports have demonstrated that certain *Thermoanaerobacter* spp. possess the inherent ability to uptake DNA (35). Nevertheless, there is a significant difference in the natural DNA uptake ability between *T. kivui* and other *Thermoanaerobacter* spp., such as *T. ethanolicus* JW200 (35). It has been reported that the transformation efficiency of *T. kivui* was much lower than that of *T. ethanolicus* JW200 (16). Several factors may affect transformation efficiency. One possible explanation for the observed low efficiency is the instability of the pMU131 derivatives in *T. kivui* (36).

### Gene knockout

This study aims to establish a CRISPR-based genome editing system within *T. kivui*. Certain genes are indispensable for the growth of this organism. For example, the HDCR-encoding gene in *T. kivui* was subjected to deletion. The resultant mutant was unable to grow on any other substrate (sugars, mannitol, or pyruvate), except when formate was added. For another example, all attempts to generate a CODH/ACS deletion mutant failed.

Previous reports have shown that the absence of the enzyme function of lactate dehydrogenase or alcohol dehydrogenase is not fatal for *T. ethanolicus* (37, 38). Thus, a gene encoding alcohol dehydrogenase (*adh*) and a gene encoding lactate dehydrogenase (*ldh*) from *T. kivui* were selected as target genes for deletion. Initially, a gene knockout experiment was performed to assess the validity and efficiency of the thermostable Cas9-based genome editing system in *T. kivui*. The *T. kivui* genome harbors two genes that code for alcohol dehydrogenase. The two genes encoding alcohol dehydrogenase are annotated as TKV_c02600 and TKV_c22260.

In this experimental design, the gene *adh* (TKV_c02600) was selected for gene knockout. A single plasmid approach using Cas9-mediated HR was developed for genome editing in *T. kivui*. Based on the PAM sequence of 5′-NNNNCGAA-3′, a PAM sequence in the *adh* gene was selected, and a 21 nt *adh* gene-targeting spacer was further designed (see Fig. S1 in the supplemental material). The tracrRNA and the crRNA were joined by using a GRAA tetraloop to generate a single-guide RNA (sgRNA) as described by Harrington et al. (30).

**Fig 1:**
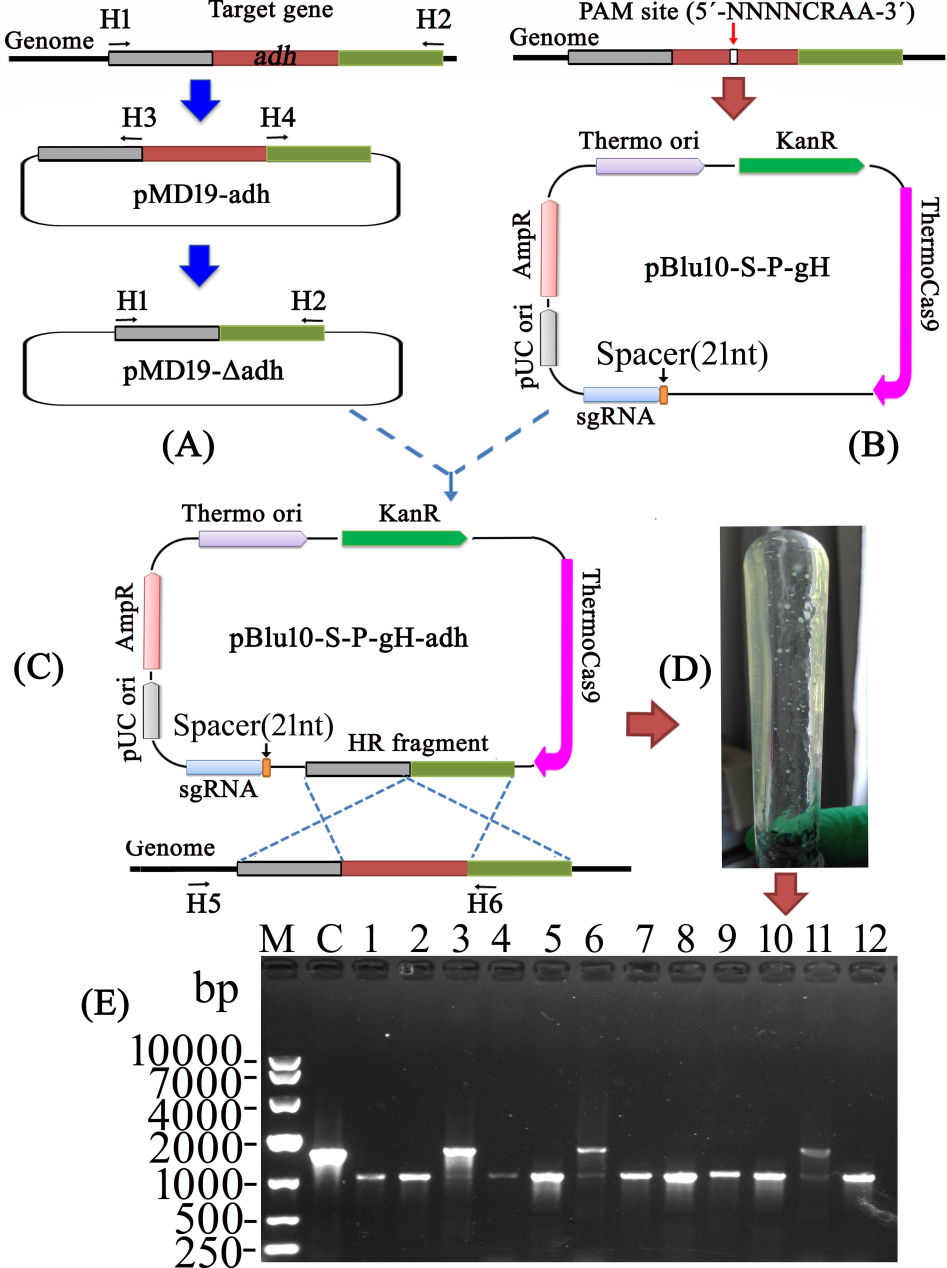
Schematic overview of thermostable Cas9-based partial *adh* gene deletion. (**A**) Construction of *adh* donor DNA containing upstream and downstream regions flanking the *adh* gene. (**B**) Construction of plasmids containing 21 nt *adh* gene-targeting spacer. (**C**) Construction of editing plasmids containing donor DNA and Cas9, sgRNA expression module. (**D**) Transformation and screening, pBlu10-S-P-gH-adh was transformed into *T. kivui* by natural competence. Single colonies were picked up and analyzed by a bacterial colony PCR to confirm the desired mutation. (**E**) Agarose gel (1%) was used to check the PCR products. Primer H5 was designed for screening and located in the upstream of the *adh* donor DNA. Thus, only the target DNA could be selectively amplified from genomic DNA but not from the plasmid pBlu10-S-P-gH-adh. The deletion of 550 bp of the partial *adh* gene permitted the amplification of an approximately 1,020 bp DNA fragment from Δ*adh* genomic DNA using primers H5 and H6. An approximately 1,560 bp fragment could be amplified from wild-type *T. kivui* genomic DNA using primers H5 and H6. Colonies (1, 2, 4, 5, 7, 8, 9, 10, and 12) were clean Δ*RSP* mutants. Colonies (3, 6, and 11) were mixed wild type/Δ*adh* genotype. Lanes: M, DNA marker; C, control; PCR amplification was carried out using wild type as the template. Lanes 1–12: PCR amplification was carried out using isolate as the template.

The plasmid named pBlu10-S-P-gH-adh was constructed to contain three essential elements: the *adh* donor DNA, sgRNA, and the thermostable Cas9 expression module. The plasmid pBlu10-S-P-gH-adh was then introduced into *T. kivui* through natural competence transformations to provide a DNA editing template for deleting approximately 550 bp of the partial *adh* gene (Fig. 1). Cultures mixed with plasmids were grown at 65°C without kanamycin sulfate for about 10 hours. Subsequently, approximately 300 µL of cultures were then plated onto solid medium containing 200 mg/L kanamycin sulfate. To confirm the partial deletion of the *adh* gene, 12 colonies were selected for analysis. Out of the 12 colonies, 9 run on the gel exhibited a knockout genotype rather than the wild-type genotype, and the other 3 displayed a mixed wild-type/Δ*adh* genotype (Fig. 1E).

A further strategy has been implemented for the selection of mutants. The mixtures of cultures and plasmids were incubated at 65°C for about 10 hours. Subsequently, up to 300 µL of transformation cultures was injected into 5 mL liquid medium containing 200 mg/L kanamycin sulfate and incubated at 65°C for about 3 days. The editing mutant may have been undergoing plasmid curing during this process. Consequently, the cultures were plated on solid medium lacking kanamycin sulfate. Picked colonies were analyzed by PCR using the primer pairs H5 and H6. It was found that the ratio of positive mutants out of all colonies obtained could reach 90%.

pBlu10-S-adh, a plasmid containing homology arms and the Cas9 expression module but no gRNA, was constructed. The plasmid pBlu10-S-adh was introduced into *T. kivui*, and 10 colonies were selected for verification by PCR using the primer pairs H5 and H6. All 10 colonies were shown to retain the *adh* gene.

**Fig 2:**
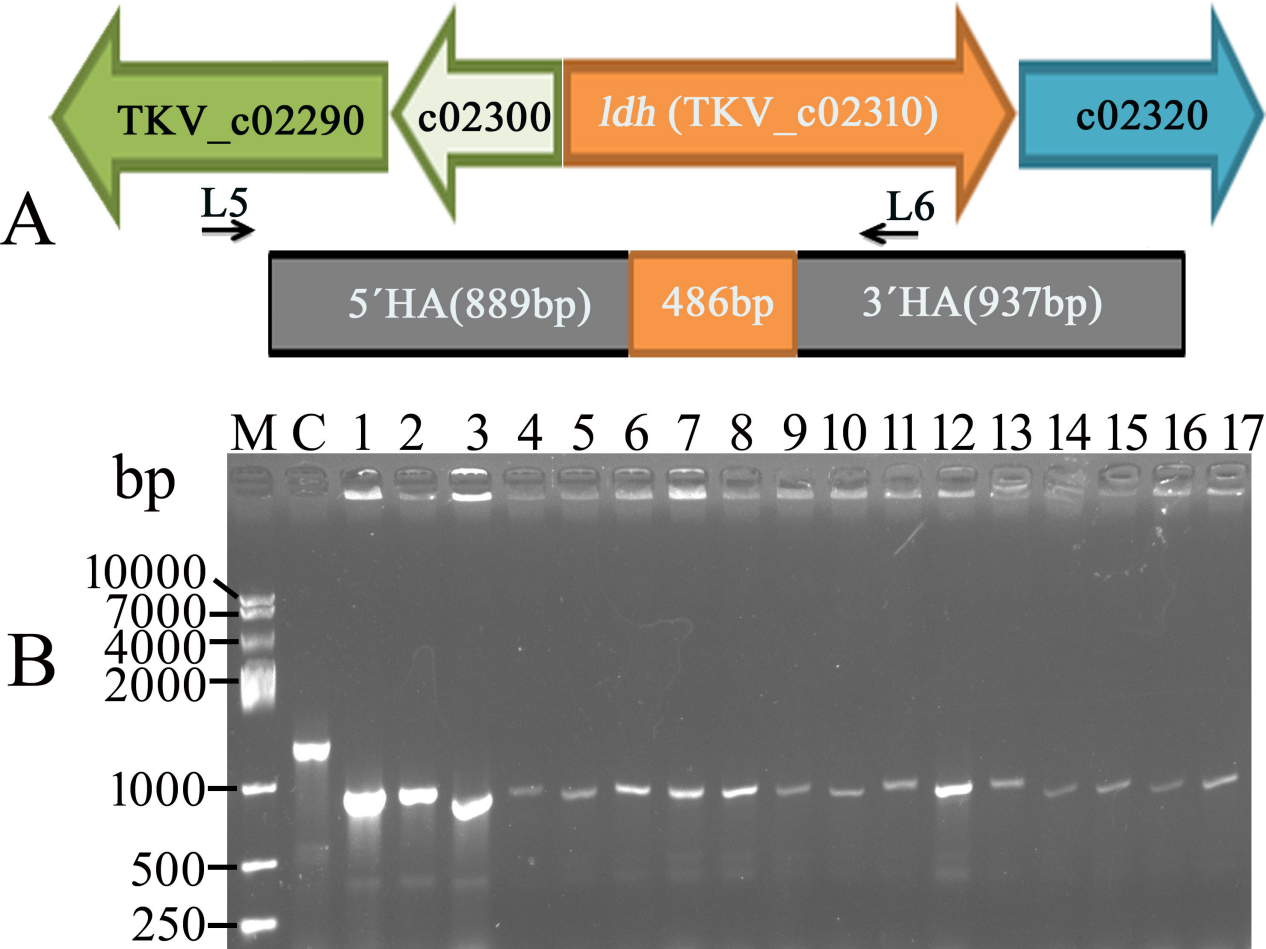
Partial *ldh* gene deletion. (**A**) Schematic overview of the design for partial *ldh* gene deletion. (**B**) Agarose gel electrophoresis showing the resulting products of genome-specific PCR on colonies from the thermostable Cas9-based *ldh gene* deletion process from the *T. kivui* genome. Primer L5 was located ahead of the upstream flanking region of the *ldh* donor DNA. Primer L6 was located in the downstream flanking region. Thus, only the target DNA could be selectively amplified from genomic DNA but not from the plasmid pBlu10-S-P-gL-ldh using primers L5 and L6. Seventeen colonies were selected and verified by PCR. All colonies were identified as clean Δ*ldh* mutants. Lane C, control; PCR amplification was carried out by using wild-type *T. kivui* as the template. Lanes 1–17: PCR amplification was carried out using Δ*ldh* mutant as the template.

Furthermore, the *ldh* gene, which codes for lactate dehydrogenase, was chosen for gene deletion. Based on the PAM sequence of 5′-NNNNCAAA-3′, pBlu10-S-P-gL-ldh was constructed to contain elements: *ldh* donor DNA, sgRNA, and the thermostable Cas9 expression module. To verify the partial gene *ldh* deletion, 17 colonies were selected for PCR analysis. All 17 colonies obtained were confirmed as clean Δ*ldh* mutants. As shown in Fig. 2, a DNA fragment of approximately 950 bp was amplified using the Δ*ldh* mutant as a template, attributable to the deletion of a DNA fragment of approximately 486 bp. In contrast, a fragment of approximately 1,440 bp was amplified from wild-type *T. kivui* genomic DNA using primers L5 and L6. DNA sequencing results further confirmed the presence of a partial deletion, along with the insertion of *Stu* I and *Eco*R I restriction enzyme sites.

Meanwhile, *T. kivui* cells were transformed with the control plasmid pBlu10-sgL-ldh. The control plasmid pBlu10-sgL-ldh contained only two essential elements, a homologous recombination template for repair and a 21 nt *ldh* gene-targeting spacer followed by an sgRNA expression module. No homologous recombination colonies were detected using the control plasmid pBlu10-sgL-ldh, which lacked the Cas9 gene expression element.

### Gene knock-in

To further assess gene integration within *T. kivui* via this system, we selected the *adhE* gene, which encodes bifunctional aldehyde/alcohol dehydrogenase (AdhE) from *T. ethanolicus* JW200, for gene knock-in using this system.

Amino acid sequence alignment analysis (https://blast.ncbi.nlm.nih.gov/Blast.cgi) showed that the alcohol dehydrogenase (TKV_c22260) from *T. kivui* exhibited 99.43% sequence identity with the alcohol dehydrogenase adhB from *T. ethanolicus* JW200. Conversely, a second alcohol dehydrogenase (TKV_c02600) from *T. kivui* displayed only 31.43% sequence identity with the alcohol dehydrogenase adhA from *T. ethanolicus* JW200. Despite possessing two genes encoding alcohol dehydrogenase, *T. kivui* is the only *Thermoanaerobacter* sp. that lacks the capability to produce ethanol (8). Therefore, the *adh* (TKV_c02600) from *T. kivui* was employed as the integration locus for *adhE* gene.

**Fig 3:**
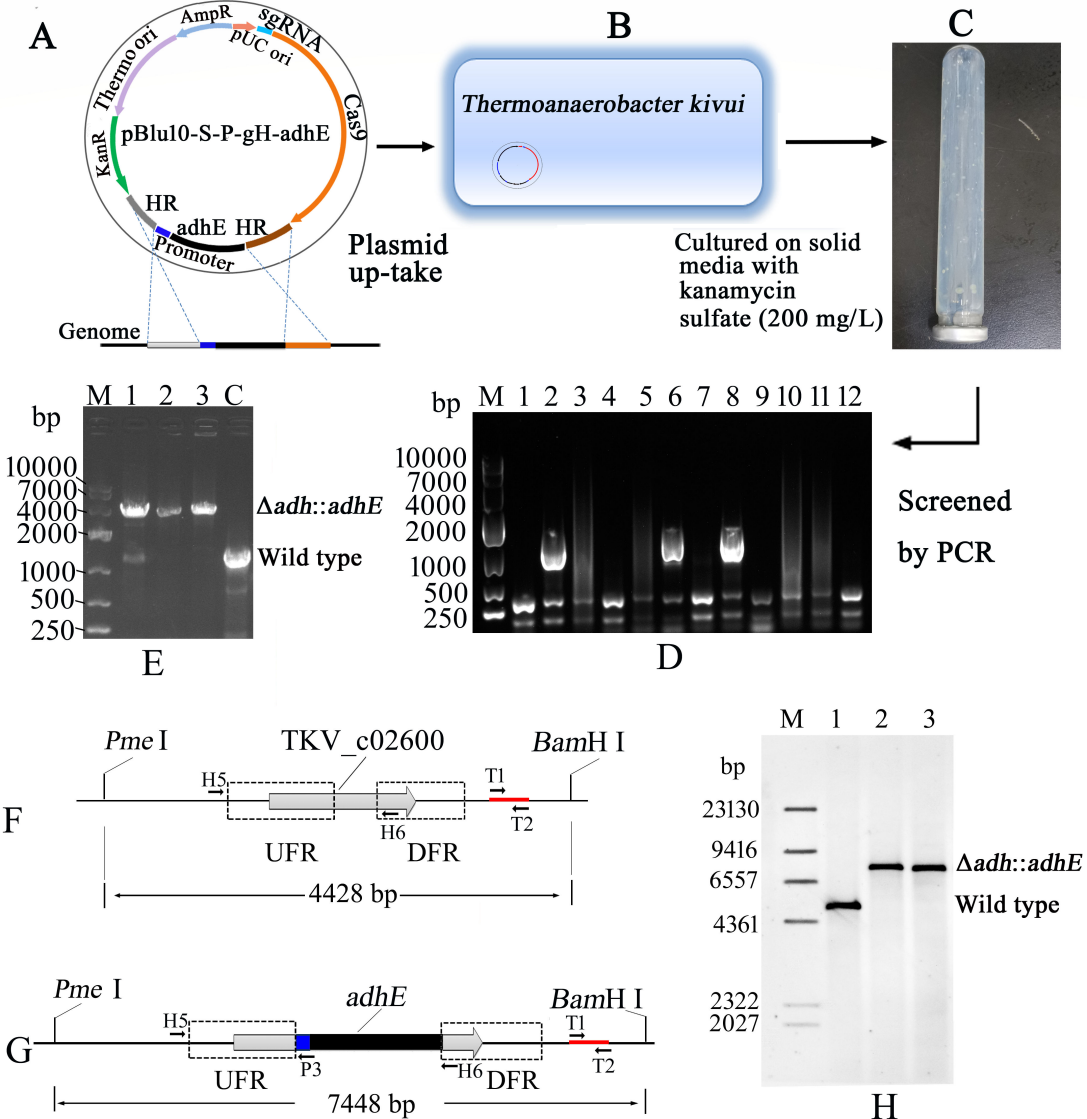
Schematic overview of thermostable Cas9-based *adhE* gene integration. (**A**) Integration plasmid pBlu10-S-P-gH-adhE contained *adh* donor DNA, *adhE* expression module, Cas9, and sgRNA expression module. (**B**) Transformation of plasmid pBlu10-S-P-gH-adhE. (**C**) Colonies appeared on solid media with kanamycin sulfate (200 mg/L). (**D**) The mutants were detected by PCR using primers H5 and P3. A fragment of about 1,200 bp was amplified from isolates 2, 6, and 8, respectively. (**E**) Lane C, control; PCR amplification was performed using wild-type *T. kivui* genomic DNA as template. Lanes 1–3: PCR amplification was carried out using genomic DNA derived from isolates 2, 6, and 8 as the template with primers H5 and H6. About 1,500 bp fragment was amplified from wild-type *T. kivui* genomic DNA (lane C). (**F**) Schematic overview of the restriction site in wild type. A DNA probe approximately 500 bp in length was amplified using primers T1 and T2. In the schematic, the binding site of the probe was indicated by a red line segment. (**G**) Schematic overview of the restriction site in Δ*adh::adhE*. The binding site of the probe was indicated by a red line segment. (**H**) Southern blot analysis of the *adh* locus with *Pme* I and *Bam*H I-digested DNA from *T. kivui* (wild type; expected fragment size: 4,482 bp) and the Δ*adh::adhE* strains isolate 6 and isolate 8 (expected fragment size: 7,448 bp). Lane M: DNA marker (labeled by digoxigenin); lane 1: *T. kivui* (wild type); lane 2: Δ*adh::adhE* strain isolate 6; lane 3: Δ*adh::adhE* strain isolate 8. DFR, downstream flanking region; UFR, upstream flanking region.

Firstly, we inserted the *adhE* gene into the shuttle expression vector pBlu10-Slay. The resulting plasmid, pBlu10-Slay-adhE, contained the Slay promoter from *T. ethanolicus* JW200, the *adhE* gene, and a terminator. Next, we fused the *adhE* gene expression element between the upstream and downstream regions flanking the *adh* gene. Subsequently, the resulting plasmid, pBlu10-S-P-gH-adhE, was introduced into *T. kivui* to serve as a DNA editing template for the integration of the *adhE* gene. PCR amplification using picked colonies as a template confirmed the correct DNA integration. To facilitate verification using colony PCR, primers (H5 and P3) have been designed for screening (Fig. 3). Primer H5 was located ahead of the upstream flanking region (UFR) of the *adh* donor DNA, and the wild-type genomic DNA lacks the sequence of primer P3. Thus, only the target DNA could be selectively amplified from mutant genomic DNA but not from wild-type genomic DNA or the plasmid pBlu10-S-P-gH-adhE. Twelve colonies were selected for verification by PCR, and the target fragment was amplified from isolates 2, 6, and 8.

The mixed wild type/Δ*adh::adhE* genotype could also be amplified using primers H5 and P3. The genomes of isolates 2, 6, and 8 were extracted and used as templates for PCR with primers H5 and H6 for further verification. Since an approximately 3,000 bp fragment containing the *adhE* expression cassette was inserted, the UFR was approximately 1,000 bp. Consequently, a fragment of about 4,000 bp could be amplified from the mutant strain. As shown in Fig. 3E, isolate 2 was a mixed wild type/Δ*adh::adhE* genotype. In contrast, isolates 6 and 8 displayed clean Δ*adh::adhE* genotypes.

Gene integration was further verified by Southern blot analysis of *Pme* I and *Bam*H I-digested genomic DNA from the wild type and isolates 6 and 8. As illustrated in Fig. 3F and G, the probe was designed to bind immediately downstream of the *adh* donor DNA flanking region. As shown in Fig. 3H, an expected fragment size of 7,448 bp was observed in the Δ*adh::adhE* genotype, while an expected fragment size of 4,428 bp appeared in the wild-type strain.

Previous research has shown that three alcohol dehydrogenases, namely, AdhE, AdhA (primary alcohol dehydrogenase), and AdhB (secondary alcohol dehydrogenase), are the key enzymes in the ethanol metabolism of *T. ethanolicus* JW200 (38). Further studies have shown that adhE-adhB or adhE-adhA is essential for ethanol production in *T. ethanolicus* JW200 (38). As a bifunctional alcohol dehydrogenase, AdhE from *T. ethanolicus* has nearly little acetaldehyde reduction activity and largely acetyl-CoA reduction activity (38). However, an interesting result has shown that the allegedly bifunctional enzyme AdhE individually is able to produce ethanol from acetyl-CoA *in vivo*, though at a lower level than the wild-type (38). A possibility was provided that the acetaldehyde reduction activity of AdhE was activated *in vivo* (38).

**Fig 4:**
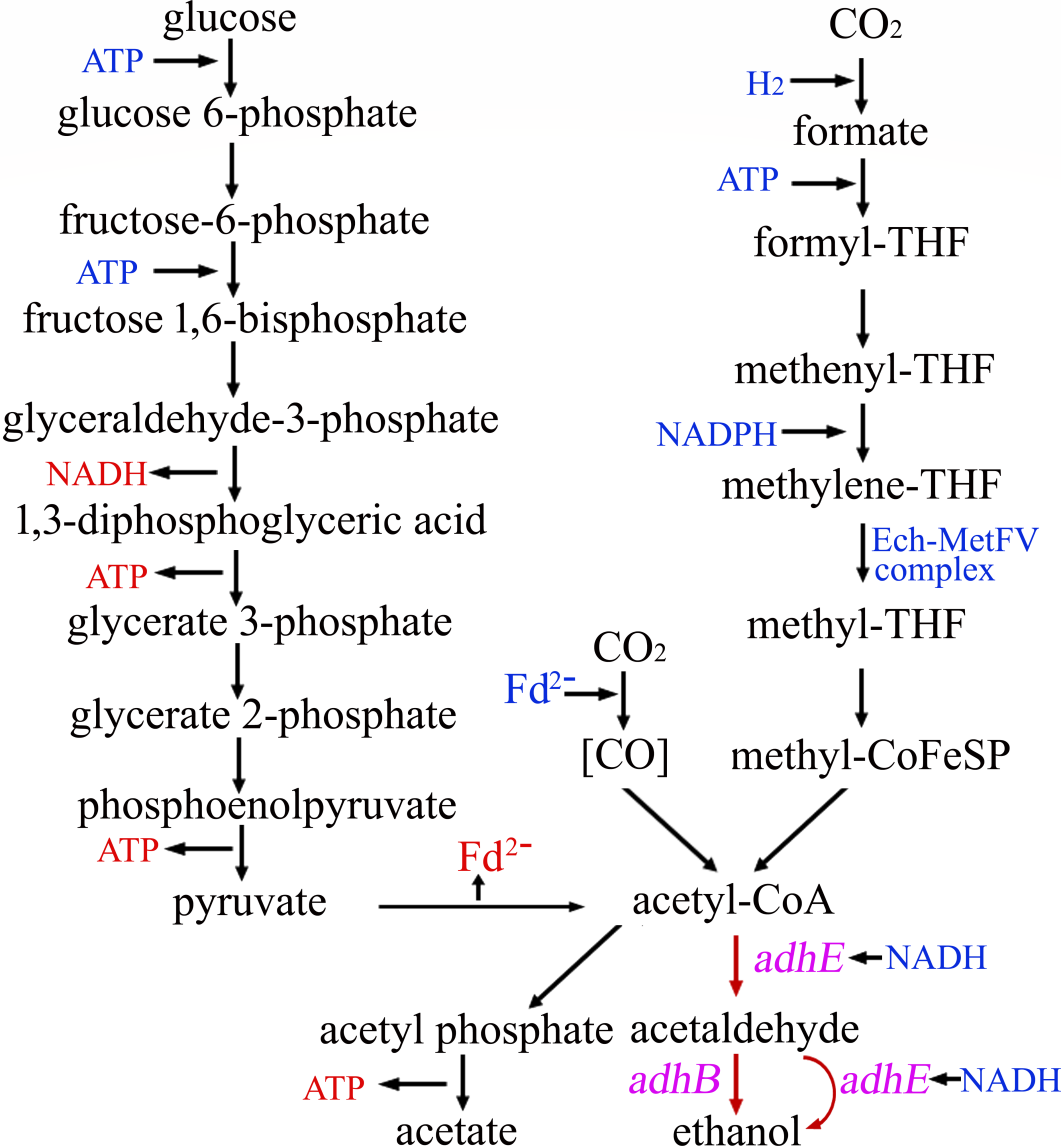
Reconstruction of ethanol production pathways in *T. kivui* via thermostable Cas9-based *adhE* gene integration. The *T. ethanolicus* JW200-derived *adhE* gene was integrated into the *T. kivui* genome to engineer a metabolic pathway for ethanol production from acetyl-CoA. CoFeSP, corrinoid/FeS protein; Ech, energy-converting hydrogenase; MetFV, methylene-THF reductase; THF, tetrahydrofolic acid.

In our experimental design, the alcohol dehydrogenase (TKV_c22260) from *T. kivui* with 99.43% identity to the adhB from *T. ethanolicus* JW200 was reserved. Another alcohol dehydrogenase (TKV_c02600) from *T. kivui* was deleted and used as the integration locus for *adhE*. A schematic representation of the ethanol biosynthesis pathways in the Δ*adh::adhE* strain has been summarized, based on the electron carriers involved in autotrophic and heterotrophic acetogenesis (13). Thus, as illustrated in Fig. 4, following the integration of the *adhE* gene into the *T. kivui* genome, ethanol biosynthesis in the M003 strain (Δ*adh::adhE*) involves enzymes that may be either solely *adhE* or a combination of *adhE* and *adhB*.

The edited cells, which contain thermostable Cas9-based gene deletion plasmids, may potentially influence cell growth. Furthermore, it is essential to remove the gene deletion plasmids from the edited cells in order to allow for subsequent metabolic engineering steps. Therefore, the edited cells were cultivated in a fresh medium for multiple passages without kanamycin sulfate. Approximately 99% of the colonies after the seventh passages were found to be sensitive to kanamycin sulfate, and this was further verified by PCR.

Ethanol production was measured in M001 (Δ*adh*), M002 (Δ*ldh*), and M003 (Δ*adh::adhE*) mutants as well as the wild-type strain under anaerobic fermentation conditions using glucose as the substrate with carbonate supplementation. High-performance liquid chromatography (HPLC) was utilized for the quantification of fermentation products derived from both the engineered strains and the wild-type strain. Ethanol accumulation was detected in the M003 (Δ*adh::adhE*) strain, while the wild-type strain, M001 (Δ*adh*), and M002 (Δ*ldh*) did not demonstrate ethanol production (Table 1). Moreover, the acetate production of the M003 (Δ*adh::adhE*) strain was significantly reduced compared to the wild-type *T. kivui*, M001 (Δ*adh*), and M002 (Δ*ldh*).

**TABLE 1:** Fermentation profile of the wild-type *T. kivui* and mutant strains using glucose as the substrate with carbonate supplementation^*a*^

Strain	Genotype	Acetate (μmol/mL)	Ethanol (μmol/mL)	Maximal OD_600_
*T. kivui*	Wild type	51.4 ± 4.1	0	2.2 ± 0.2
M001	Δ*adh*	50.7 ± 2.1	0	2.1 ± 0.1
M002	Δ*ldh*	50.2 ± 1.4	0	2.1 ± 0.1
M003	Δ*adh*::*adhE*	23.8 ± 3.1	17.3 ± 3.3	2.5 ± 0.1

^*a*^ Fermentations were performed in 10 mL of prepared medium containing 0.5% glucose and 0.58% KHCO_3_ at 65°C. Three replicates were performed for each sample.

**Fig 5:**
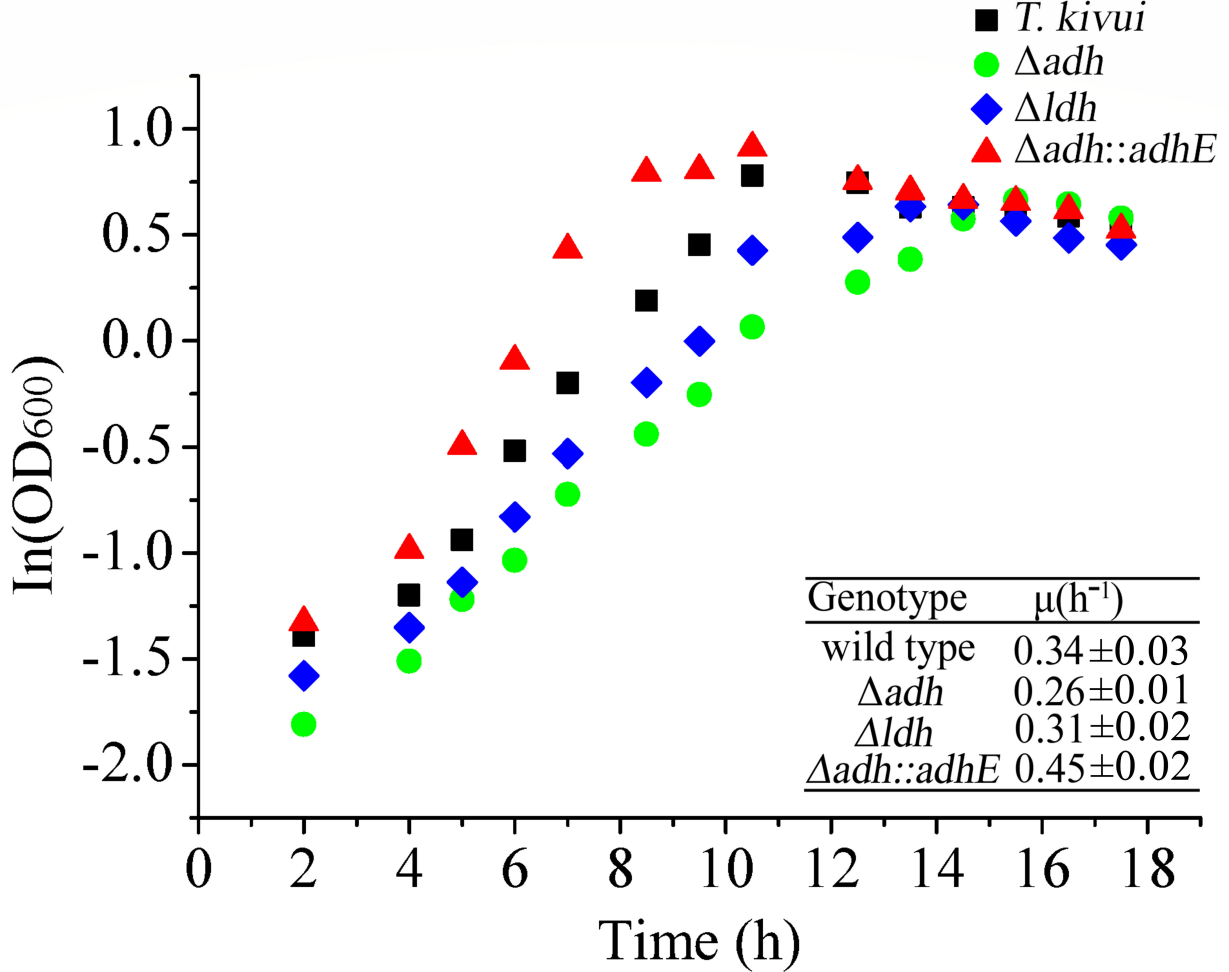
Growth of wild-type *T. kivui* (filled squares), Δ*adh* (filled circles), Δ*ldh* (filled diamonds), and Δ*adh::adhE* (filled triangles) on glucose in the presence of carbonate in medium at 65℃. Inset: specific growth rates (μ). Shown is one representative experiment out of three independent biological replicates.

In addition, the growth curves of these strains were also assessed. Specific growth rates (*μ*) were determined using the slopes of the growth curves (semi-logarithmic plots) in the exponential phase. As shown in Fig. 5, specific growth rates (*μ*) of the wild-type strain, Δ*adh*, Δ*ldh*, and Δ*adh::adhE* were 0.34 ± 0.03 h^−1^ (*R*^2^ = 0.9959), 0.26 ± 0.01 h^−1^ (*R*^2^ = 0.9968), 0.31 ± 0.02 h^−1^ (*R*^2^ = 0.9944), and 0.45 ± 0.02 h^−1^ (*R*^2^ = 0.9974), respectively. It was observed that the growth of Δ*adh* and Δ*ldh* remained markedly slower than that of wild-type *T. kivui* (Fig. 5). It is noteworthy that M003 (Δ*adh::adhE*) exhibits superior growth compared to the wild type, M001 (Δ*adh*), and M002 (Δ*ldh*), reaching higher cell densities (Fig. 5). One possible explanation for this alteration is the reduction in acetate concentrations, which could influence the media’s pH. Alternatively, the faster growth rate observed in M003 (Δ*adh::adhE*) may stem from more efficient electron recycling, even though ethanol production yields less ATP. Further investigation will be necessary to elucidate these mechanisms. Although the ethanol productivity of strain M003 (Δ*adh::adhE*) remains low, the CRISPR/Cas9 system has successfully demonstrated the integration of genes into the *T. kivui* genome.

In general, the efficiency of CRISPR/Cas-based genome editing depends on several factors, including the expression levels of both gRNA and Cas9 and the efficiency of homologous recombination, among others (31, 39). Recently, a fluorescent reporter system was established, and plasmid instability was observed in *T. kivui* (36). A previous report suggested that pMU131 derivatives are not segregationally stable in *T. kivui*, with stability sharply decreasing with increasing temperatures (36). To enhance plasmid stability, antibiotic pressure was required (36). During our plasmid curing experiment, the mutants were transferred to a fresh medium without kanamycin sulfate and cultivated at 65°C. Our results have also shown that the editing plasmids could be cured conveniently from edited cells after several passages. This approach will facilitate the manipulation of multigene editing for *T. kivui* in future studies. To facilitate continuous genome editing, strategies for curing Cas9 and gRNA expression plasmids have been reported in the literature (40). For example, the editing *Corynebacterium glutamicum* cells were serially transferred into fresh medium without antibiotics with the objective of eliminating the Cas9 and gRNA expression plasmids from edited cells (41). In our experiment, a comparable methodology was employed to eliminate the editing plasmids.

In the previous genetic system using *pyrE* as a selective marker for markerless deletions on the *T. kivui* chromosome, two rounds of selection were usually required (16). After the second round of selection, two out of three isolates were clean and markerless deletion mutants (16). A novel genome editing tool, Hi-TARGET, has recently been developed based on the endogenous CRISPR Type I-B system from *T. kivui*. This system achieves perfect editing efficiency (100%) for both gene knockout and knock-in, and 49% efficiency for creating point mutations (42). In our experiment, the gene deletion efficiency for *ldh* was 100%, and the gene deletion efficiency for *adh* was 90%. Gene integration efficiency for a fragment of about 3,000 bp was approximately 17%. The Cas9-based genome editing system developed for *T. kivui* provides a simple and rapid method for mutant isolation, representing a significant expansion of its genetic toolbox.

In conclusion, a thermostable Cas9-based genome editing tool has been successfully developed for the thermophilic acetogen *T. kivui*. The efficacy of both gene knockout and knock-in has been demonstrated, establishing this genetic system as a powerful tool for elucidating the physiological functions of enzymes involved in *T. kivui*’s energy and carbon metabolism. Furthermore, this technology enables the metabolic engineering of *T. kivui* for the production of diverse designer chemicals.

## MATERIALS AND METHODS

### Strain and cultivation

*Escherichia coli* JM109 was used for gene cloning and cultivated aerobically in Luria–Bertani medium at 37°C. The antibiotic ampicillin (100 mg/L) was added to the medium as required. *T. kivui* (DSM2030), obtained from Deutsche Sammlung von Mikroorganismen und Zellkulturen, Germany, was routinely cultivated under strict anaerobic conditions at 65°C. Complex medium was prepared as described previously (1), containing per liter 5.8 g KHCO_3_, 0.22 g KH_2_PO_4_, 0.22 g K_2_HPO_4_, 4.5 g NaH_2_PO_4_, 6.1 g Na_2_HPO_4_·12H_2_O, 0.31 g NH_4_Cl, 0.22 g (NH_4_)_2_SO_4_, 0.45 g NaCl, 0.09 g MgSO_4_·7H_2_O, 0.001 g CaCl_2_, 0.00013 g FeSO_4_·7H_2_O, 4.5 g yeast extract, 0.002 g resazurin, and 5 g glucose. The medium was flushed with N_2_ before autoclaving. Cultures were incubated at 65°C under anaerobic conditions. Agar medium was supplemented with 2% agar. The antibiotic kanamycin sulfate (200 mg/L) was added to the medium as required.

### Molecular biology experiment

All general molecular biology procedures were carried out according to standard procedures (43). Genomic DNA extraction Kit (B518225) and Plasmid Extraction Kit (B518191) were purchased from Sangon Biotech (Shanghai, China). All primers used in this study are summarized in Table 2 and the plasmids used in this study are listed in Table 3.

**TABLE 2:** Primers used in this study

Primer	Sequence (5′−3′)
H1	AAACTCGAGTAAATTCAACTGGCTTACGTC
H2	GATGGATCCGCATTATTGCGATCATAAAATC
H3	ATCGAAACCCTACTTTGAACCATC
H4	TTTGATATCGCTCAAAGAAGCAGGCATTTC
PgH1	AGGTCCACATATCTTAATTTTATCACTATAATATTG
PgH2	ACCTTATGAAAGTCATAGTTCCCCTGAGAAATC
C1	CTTTAATTTTATCACTATAATATTG
H5	AATTGTCGGGATAGTATTTATC
H6	TCAGAAATGCCTGCTTCTTTG
L1	AAACTCGAGACGAGCATAAGTGATAATGTC
L2	GATGGATCCTTTGACTCCATACTCTCAATG
L3	GTTAGGCCTTCTTGCTCATAATATCTTCTC
L4	TTTGAATTCACGGATATATAATTGGAGAAC
L5	GCACAGTTTTAAACTGCATTG
L6	CATGTTCTCCAATTATATATC
L7	AACATTCTGTTCCAAGGGTATC
L8	GTAGAGGTAGCGTATACTACTG
PgL1	AAAATCATTGTATCTTAATTTTATCACTATAATATTG
PgL2	TAAAGACATTGTCATAGTTCCCCTGAGAAATC
P1	TTTAGGCCTCATATCACAGTCAATCCTCCTC
P2	CAAGGTCTTTTTTTCTAGATAGTAC
P3	TCAATCCTCCTCCTTGTATTTG
E1	CCGAACTTATTACAAGAAAGAAGAG
E2	GCGTCTAGATTATTCTCCATAGGCTTTTCTATAT
E3	TCGTGAGATACCCTTGGAACAG
E4	AAACGCCTGGTATCTTTATAG
T1	GGTTGTGATGGATTGGAA
T2	TAAATGTGATAGGGGTTG
K1	CAAAACAACTTTGAAAAAGC
K2	TATAGAACCT CCTGGATTAC

**TABLE 3:** Plasmids used in this study

Plasmid	Features	Reference
pMD19-T	T-vector, TA-cloning	Takara-Bio, Dalian, China
pBlu10-S-P-sgT	Containing Cas9 gene expression elements, 21 nt *Tdk* gene-targeting spacer, and sgRNA expression module under the control of Pat promoter	(33)
pBlu10-Slay-Cas9	Containing Cas9 gene expression elements	(33)
pMD19-adh	Containing 5′ and 3′ flanking fragments and the full-length *adh* gene	This work
pMD19-Δadh	Containing 5′ and 3′ flanking fragments and partial *adh* segments	This work
pBlu10-S-P-gH	Containing Cas9 gene expression elements, 21 nt *adh* gene-targeting spacer, and sgRNA expression module	This work
pBlu10-S-adh	Containing Cas9 gene and *adh* donor DNA	This work
pBlu10-S-P-gH-adh	Containing Cas9 gene, sgRNA expression elements, 21 nt *adh* gene-targeting spacer, and *adh* donor DNA	This work
pMD19-ldh	Containing 5′ and 3′ flanking fragments and the full-length *ldh* gene	This work
pMD19-Δldh	Containing 5′ and 3′ flanking fragments and partial *ldh* segments	This work
pBlu10-S-P-gL	Containing Cas9 gene expression elements, 21 nt *ldh* gene-targeting spacer, and sgRNA expression module	This work
pBlu10-S-P-gL-ldh	Containing Cas9 gene, sgRNA expression elements, 21 nt *ldh* gene-targeting spacer, and *ldh* donor DNA	This work
pBlu10-sgL-ldh	Containing sgRNA expression elements, 21 nt *ldh* gene-targeting spacer, and *ldh* donor DNA	This work
pBlu10-Slay	Shuttle expression vector containing Slay promoter, multicloning sites (*Stu*I and *Xba*І) and a terminator	This work
pBlu10-Slay-adhE	Expression vector containing *adhE* gene expression elements	This work
pMD19-Δadh-adhE	Containing 5′ and 3′ flanking fragments and *adhE* gene expression elements	This work
pBlu10-S-P-gH-adhE	Containing Cas9 gene expression element, 21 nt *adh* gene-targeting spacer, sgRNA expression module, *adh* donor DNA and *adhE* gene expression elements	This work

### Transformation of *T. kivui*

DNA uptake by *T. kivui* was routinely performed by natural competence as described by Basen et al. (16). In brief, 300 µL subcultures were mixed with 1 µg plasmid DNA in a Hungate anaerobic culture tube and grown at 65°C under anaerobic conditions for several hours. In this experiment, approximately 5 mL of solid medium was encapsulated within a Hungate tube (18 mm in diameter and 150 mm in length). Following the sterilization process, the tube was cooled and then placed in a water bath at a temperature of 65°C. For the selection of mutants, up to 300 µL of the subcultures was injected into one tube containing solid media. Kanamycin was added, if necessary, at a concentration of 200 mg/L. A total of 10 tubes containing solid medium were used. Thereafter, the tubes were taken out and rolled on a tabletop to facilitate the adhesion of the culture medium to the tube wall and subsequently cultured at 65°C in an incubator.

### Deletion of the *adh* gene

All primer sequences and plasmids used in this study are listed in Tables 2 and 3, respectively. Molecular manipulations, including DNA cloning and transformation in *E. coli*, were performed according to standard protocols. Initially, the 5′ and 3′ flanking fragments containing the *adh* gene (TKV_c02600) were amplified from *T. kivui* genomic DNA via PCR using Ex Taq DNA Polymerase (Takara-Bio, Dalian, China) with primers H1 and H2 (Table 2). The amplified fragments were inserted into a T-Vector (pMD19 T Vector Cloning Kit, Takara-Bio) to construct vector pMD19-adh. The resulting vector pMD19-adh was used as template with the primers H3 and H4 for PCR. The PCR products were phosphorylated with T4 Polynucleotide Kinase (Takara-Bio) and then ligated with T4 DNA ligase (Takara-Bio) to generate plasmid pMD19-Δadh.

In our previous study, plasmid pBlu10-S-P-sgT (33) contained Cas9 gene expression elements, sgRNA expression module, a thermostable gram-positive origin of replication from plasmid pMU131, and a thermostable kanamycin resistance gene (Htk). Based on the *adh* (TKV_c02600) sequence, the 21 nt *adh* gene-targeting spacer was amplified with primers pgH1 and pgH2 using pBlu10-S-P-sgT as template. The PCR products were phosphorylated with T4 Polynucleotide Kinase (Takara-Bio) and then ligated with T4 DNA ligase to generate plasmid pBlu10-S-P-gH (Table 3). The sequence of the plasmid pBlu10-S-P-gH, including a clear annotation of each element, has been provided in the supplemental material.

The *adh* donor DNA was amplified with PrimeSTAR HS DNA Polymerase (Takara-Bio) using primers H1 and H2 and pMD19-Δadh as template. The PCR fragments were digested with *Bam*H І and *Xho* І and subcloned into the *Bam*H І and *Xho* І sites of pBlu10-S-P-gH. The resulting gene deletion plasmid was referred to as pBlu10-S-P-gH-adh. The plasmid pBlu10-S-P-gH-adh was retrieved from *E. coli* JM109 and transformed into *T. kivui*. Colonies that appeared on the solid medium with 200 mg/L kanamycin sulfate were selected. The picked colonies were analyzed by PCR amplification, and the Δ*adh* mutant strain was named M001. Multiple repeated experiments were performed to verify the reproducibility.

A control plasmid pBlu10-S-adh containing homology arms and Cas9 but lacking gRNA was constructed. The plasmid pBlu10-S-P-gH-adh was used as a template with primers PgH2 and C1. The PCR products were phosphorylated with T4 Polynucleotide Kinase (Takara-Bio) and then ligated with T4 DNA ligase to generate plasmid pBlu10-S-adh.

### Deletion of the *ldh* gene

The 5′ and 3′ flanking fragments containing the *ldh* gene (TKV_c02310) were amplified from *T. kivui* genomic DNA via PCR with Ex Taq DNA Polymerase using primers L1 and L2 (Table 2). PCR products were inserted into the T-Vector (pMD19 T Vector Cloning Kit, Takara-Bio) to construct vector pMD19-ldh. Then pMD19-ldh was used as template with primers L3 and L4 for PCR. The PCR products were phosphorylated with T4 Polynucleotide Kinase (Takara-Bio) and then ligated with T4 DNA ligase to generate plasmid pMD19-Δldh.

Plasmid (pBlu10-S-P-sgT) was used as template with primers pgL1 and pgL2 for PCR. The PCR products were phosphorylated with T4 Polynucleotide Kinase (Takara-Bio) and then ligated with T4 DNA ligase to generate plasmid pBlu10-S-P-gL (Table 3). The *ldh* donor DNA was amplified using pMD19-Δldh as template with primers L1 and L2. The PCR products were digested with *Bam*H І and *Xho* І and subcloned into the *Bam*H І and *Xho* І sites of pBlu10-S-P-gL. The resulting gene deletion plasmid was referred to as pBlu10-S-P-gL-ldh. Plasmid pBlu10-S-P-gL-ldh was transformed into *T. kivui*. The picked colonies were analyzed by PCR amplification using primers L5 and L6 (Table 2). The Δ*ldh* mutant was named strain M002.

The plasmid pBlu10-S-P-gL-ldh was used as template with primers L7 and L8 for PCR. The PCR products were phosphorylated with T4 Polynucleotide Kinase (Takara-Bio) and then ligated with T4 DNA ligase to generate the control plasmid pBlu10-sgL-ldh.

### Integration of *adhE* from *T. ethanolicus* into *T. kivui*

PCR was performed with pBlu10-S-P-sgT (30) as templates and primers P1/P2. The PCR products were phosphorylated with T4 Polynucleotide Kinase (Takara-Bio) and then ligated with T4 DNA ligase (Takara-Bio) to generate plasmid pBlu10-Slay. The full-length *adhE* gene (GenBank: DQ836061.1) was amplified from *T. ethanolicus* genomic DNA via PCR with primers E1 and E2. The PCR fragments of the *adhE* gene were digested with *Xba* І and subcloned into the *Stu* І and *Xba* І sites of pBlu10-Slay. The resulting plasmid was referred to as pBlu10-Slay-adhE.

The sequences, including Slay promoter, *adhE* gene, and the terminator, were amplified from pBlu10-Slay-adhE using primers E3 and E4. The PCR products were then phosphorylated and ligated with the fragments amplified from pMD19-Δadh with the primers H3 and H4. The resulting vector was referred to as pMD19-Δadh-adhE.

The DNA fragment was amplified with PrimeSTAR HS DNA Polymerase (Takara-Bio) using primers H1 and H2 and plasmid pMD19-Δadh-adhE as template. The PCR products were digested with *Bam*H І and *Xho* І and subcloned into the *Bam*H І and *Xho* І sites of pBlu10-S-P-gH. The resulting plasmid was referred to as pBlu10-S-P-gH-adhE. Plasmid pBlu10-S-P-gH-adhE was transformed into *T. kivui*. The picked colonies were analyzed by PCR amplification using primers (H5 and P3). The mutant (Δ*adh::adhE*) was named strain M003.

### Plasmid curing

The method of plasmid curing was described by Liu et al. (41). In order to remove the plasmids from edited cells, the mutant was transferred into fresh medium without kanamycin sulfate and cultivated at 65°C. The culture was serially transferred into fresh medium several times without antibiotics. Colonies were confirmed as cured by determining their sensitivity to kanamycin sulfate and by means of PCR verification using primers K1 and K2.

### Analytical techniques

Strains were grown at 65°C under anaerobic conditions using glucose as substrate with carbonate supplementation. HPLC was performed to analyze ethanol and acetate as described by Le et al. (33). The column was eluted at 50°C with 0.25 g/L H_2_SO_4_ at a flow rate of 0.4 mL/min. Ethanol and acetate were detected by a RID-10A refractive-index detector (Shimadzu, Kyoto, Japan).

### Southern blot analysis

Southern blot analysis was performed by Nanjing Zoonbio Biotechnology Co., Ltd., China. The genotypes of wild type and mutants were analyzed by hybridization with digoxigenin (DIG)-labeled DNA according to standard procedures (42). Nucleic acid labeling (Roche) was used for the generation of the DIG-labeled DNA probe. The genomes were digested with restriction enzymes *Pme* I and *Bam*H I. DNA fragments were separated on a 0.7% agarose gel and subsequently transferred to a charged nylon membrane (HyBond *N*^+^, Amersham Corporation). The membrane was hybridized with the probe at 37°C overnight. Detection was performed by incubating the membrane in CSPD substrate (Roche) for 5 min at 25°C.

## SUPPLEMENTAL MATERIAL

Supplemental material aem.01170-25-s0001.docx Figure S1 and the sequence of plasmid pBlu10-S-P-gH.

ASM does not own the copyrights to Supplemental Material that may be linked to, or accessed through, an article. The authors have granted ASM a non-exclusive, world-wide license to publish the Supplemental Material files. Please contact the corresponding author directly for reuse.

## References

[1] Leigh JA, Mayer F, Wolfe RS. 1981. Acetogenium kivui, a new thermophilic hydrogen-oxidizing acetogenic bacterium. Arch Microbiol 129:275–280. doi:10.1007/BF00414697

[2] Moon J, Henke L, Merz N, Basen M. 2019. A thermostable mannitol-1-phosphate dehydrogenase is required in mannitol metabolism of the thermophilic acetogenic bacterium Thermoanaerobacter kivui. Environ Microbiol 21:3728–3736. doi:10.1111/1462-2920.14720 31219674

[3] Moon J, Jain S, Müller V, Basen M. 2020. Homoacetogenic conversion of mannitol by the thermophilic acetogenic bacterium Thermoanaerobacter kivui requires external CO_2_ Front Microbiol 11:571736. doi:10.3389/fmicb.2020.571736 33042077 PMC7522397

[4] Schwarz FM, Müller V. 2020. Whole-cell biocatalysis for hydrogen storage and syngas conversion to formate using a thermophilic acetogen. Biotechnol Biofuels 13:32. doi:10.1186/s13068-020-1670-x 32140177 PMC7048051

[5] Burger Y, Schwarz FM, Müller V. 2022. Formate-driven H_2_ production by whole cells of Thermoanaerobacter kivui*.* Biotechnol Biofuels Bioprod 15:48. doi:10.1186/s13068-022-02147-5 35545791 PMC9097184

[6] Liu ZY, Jia DC, Zhang KD, Zhu HF, Zhang Q, Jiang WH, Gu Y, Li FL. 2020. Ethanol metabolism dynamics in Clostridium ljungdahlii grown on carbon monoxide. Appl Environ Microbiol 86:e00730-20. doi:10.1128/AEM.00730-20 32414802 PMC7357473

[7] Takemura K, Kato J, Kato S, Fujii T, Wada K, Iwasaki Y, Aoi Y, Matsushika A, Murakami K, Nakashimada Y. 2021. Autotrophic growth and ethanol production enabled by diverting acetate flux in the metabolically engineered Moorella thermoacetica*.* J Biosci Bioeng 132:569–574. doi:10.1016/j.jbiosc.2021.08.005 34518108

[8] Basen M, Müller V. 2017. “Hot” acetogenesis. Extremophiles 21:15–26. doi:10.1007/s00792-016-0873-3 27623994

[9] Katsyv A, Essig M, Bedendi G, Sahin S, Milton RD, Müller V. 2023. Characterization of ferredoxins from the thermophilic, acetogenic bacterium Thermoanaerobacter kivui*.* FEBS J 290:4107–4125. doi:10.1111/febs.16801 37074156

[10] Katsyv A, Müller V. 2022. A purified energy-converting hydrogenase from Thermoanaerobacter kivui demonstrates coupled H^+^-translocation and reduction in vitro. J Biol Chem 298:102216. doi:10.1016/j.jbc.2022.102216 35779632 PMC9356269

[11] Dietrich HM, Righetto RD, Kumar A, Wietrzynski W, Trischler R, Schuller SK, Wagner J, Schwarz FM, Engel BD, Müller V, Schuller JM. 2022. Membrane-anchored HDCR nanowires drive hydrogen-powered CO_2_ fixation. Nature 607:823–830. doi:10.1038/s41586-022-04971-z 35859174

[12] Katsyv A, Schoelmerich MC, Basen M, Müller V. 2021. The pyruvate:ferredoxin oxidoreductase of the thermophilic acetogen, Thermoanaerobacter kivui. FEBS Open Bio 11:1332–1342. doi:10.1002/2211-5463.13136 PMC8091585 33660937

[13] Katsyv A, Jain S, Basen M, Müller V. 2021. Electron carriers involved in autotrophic and heterotrophic acetogenesis in the thermophilic bacterium Thermoanaerobacter kivui. Extremophiles 25:513–526. doi:10.1007/s00792-021-01247-8 34647163 PMC8578170

[14] Schwarz FM, Schuchmann K, Müller V. 2018. Hydrogenation of CO_2_ at ambient pressure catalyzed by a highly active thermostable biocatalyst. Biotechnol Biofuels 11:237. doi:10.1186/s13068-018-1236-3 30186365 PMC6119302

[15] Katsyv A, Kumar A, Saura P, Pöverlein MC, Freibert SA, T Stripp S, Jain S, Gamiz-Hernandez AP, Kaila VRI, Müller V, Schuller JM. 2023. Molecular basis of the electron bifurcation mechanism in the [FeFe]-hydrogenase complex HydABC. J Am Chem Soc 145:5696–5709. doi:10.1021/jacs.2c11683 36811855 PMC10021017

[16] Basen M, Geiger I, Henke L, Müller V. 2018. A genetic system for the thermophilic acetogenic bacterium Thermoanaerobacter kivui. Appl Environ Microbiol 84:e02210-17. doi:10.1128/AEM.02210-17 29150512 PMC5772241

[17] Taylor MP, Eley KL, Martin S, Tuffin MI, Burton SG, Cowan DA. 2009. Thermophilic ethanologenesis: future prospects for second-generation bioethanol production. Trends Biotechnol 27:398–405. doi:10.1016/j.tibtech.2009.03.006 19481826

[18] Crosby JR, Laemthong T, Lewis AM, Straub CT, Adams MW, Kelly RM. 2019. Extreme thermophiles as emerging metabolic engineering platforms. Curr Opin Biotechnol 59:55–64. doi:10.1016/j.copbio.2019.02.006 30875665

[19] Lin L, Xu J. 2013. Dissecting and engineering metabolic and regulatory networks of thermophilic bacteria for biofuel production. Biotechnol Adv 31:827–837. doi:10.1016/j.biotechadv.2013.03.003 23510903

[20] Zeldes BM, Keller MW, Loder AJ, Straub CT, Adams MWW, Kelly RM. 2015. Extremely thermophilic microorganisms as metabolic engineering platforms for production of fuels and industrial chemicals. Front Microbiol 6:1209. doi:10.3389/fmicb.2015.01209 26594201 PMC4633485

[21] Leang C, Ueki T, Nevin KP, Lovley DR. 2013. A genetic system for Clostridium ljungdahlii: a chassis for autotrophic production of biocommodities and a model homoacetogen. Appl Environ Microbiol 79:1102–1109. doi:10.1128/AEM.02891-12 23204413 PMC3568603

[22] Bao J, de Dios Mateos E, Scheller S. 2022. Efficient CRISPR/Cas12a-based genome-editing toolbox for metabolic engineering in Methanococcus maripaludis. ACS Synth Biol 11:2496–2503. doi:10.1021/acssynbio.2c00137 35730587 PMC9295151

[23] Zhao R, Liu Y, Zhang H, Chai C, Wang J, Jiang W, Gu Y. 2019. CRISPR-Cas12a-mediated gene deletion and regulation in Clostridium ljungdahlii and its application in carbon flux redirection in synthesis gas fermentation. ACS Synth Biol 8:2270–2279. doi:10.1021/acssynbio.9b00033 31526005

[24] Huang H, Chai C, Li N, Rowe P, Minton NP, Yang S, Jiang W, Gu Y. 2016. CRISPR/Cas9-based efficient genome editing in Clostridium ljungdahlii, an autotrophic gas-fermenting bacterium. ACS Synth Biol 5:1355–1361. doi:10.1021/acssynbio.6b00044 27276212

[25] Nagaraju S, Davies NK, Walker DJF, Köpke M, Simpson SD. 2016. Genome editing of Clostridium autoethanogenum using CRISPR/Cas9. Biotechnol Biofuels 9:219. doi:10.1186/s13068-016-0638-3 27777621 PMC5069954

[26] Jain S, Dietrich HM, Müller V, Basen M. 2020. Formate is required for growth of the thermophilic acetogenic bacterium Thermoanaerobacter kivui lacking hydrogen-dependent carbon dioxide reductase (HDCR). Front Microbiol 11:59. doi:10.3389/fmicb.2020.00059 32082286 PMC7005907

[27] Jain S, Katsyv A, Basen M, Müller V. 2021. The monofunctional CO dehydrogenase CooS is essential for growth of Thermoanaerobacter kivui on carbon monoxide. Extremophiles 26:4. doi:10.1007/s00792-021-01251-y 34919167 PMC8683389

[28] Baum C, Zeldes B, Poehlein A, Daniel R, Müller V, Basen M. 2024. The energy-converting hydrogenase Ech2 is important for the growth of the thermophilic acetogen Thermoanaerobacter kivui on ferredoxin-dependent substrates. Microbiol Spectr 12:e0338023. doi:10.1128/spectrum.03380-23 38385688 PMC10986591

[29] Mougiakos I, Mohanraju P, Bosma EF, Vrouwe V, Finger Bou M, Naduthodi MIS, Gussak A, Brinkman RBL, van Kranenburg R, van der Oost J. 2017. Characterizing a thermostable Cas9 for bacterial genome editing and silencing. Nat Commun 8:1647. doi:10.1038/s41467-017-01591-4 29162801 PMC5698299

[30] Harrington LB, Paez-Espino D, Staahl BT, Chen JS, Ma E, Kyrpides NC, Doudna JA. 2017. A thermostable Cas9 with increased lifetime in human plasma. Nat Commun 8:1424. doi:10.1038/s41467-017-01408-4 29127284 PMC5681539

[31] Walker JE, Lanahan AA, Zheng T, Toruno C, Lynd LR, Cameron JC, Olson DG, Eckert CA. 2020. Development of both type I-B and type II CRISPR/Cas genome editing systems in the cellulolytic bacterium Clostridium thermocellum. Metab Eng Commun 10:e00116. doi:10.1016/j.mec.2019.e00116 31890588 PMC6926293

[32] Adalsteinsson BT, Kristjansdottir T, Merre W, Helleux A, Dusaucy J, Tourigny M, Fridjonsson O, Hreggvidsson GO. 2021. Efficient genome editing of an extreme thermophile, Thermus thermophilus, using a thermostable Cas9 variant. Sci Rep 11:9586. doi:10.1038/s41598-021-89029-2 33953310 PMC8100143

[33] Le Y, Fu Y, Sun J. 2020. Genome editing of the anaerobic thermophile Thermoanaerobacter ethanolicus using thermostable Cas9. Appl Environ Microbiol 87:e01773-20. doi:10.1128/AEM.01773-20 33067194 PMC7755235

[34] Dai K, Fu H, Guo X, Qu C, Lan Y, Wang J. 2022. Exploiting the type I-B CRISPR genome editing system in Thermoanaerobacterium aotearoense SCUT27 and engineering the strain for enhanced ethanol production. Appl Environ Microbiol 88:e0075122. doi:10.1128/aem.00751-22 35862665 PMC9361813

[35] Shaw AJ, Hogsett DA, Lynd LR. 2010. Natural competence in Thermoanaerobacter and Thermoanaerobacterium species. Appl Environ Microbiol 76:4713–4719. doi:10.1128/AEM.00402-10 20472726 PMC2901744

[36] Hocq R, Bottone S, Gautier A, Pflügl S. 2023. A fluorescent reporter system for anaerobic thermophiles. Front Bioeng Biotechnol 11:1226889. doi:10.3389/fbioe.2023.1226889 37476481 PMC10355840

[37] Shao X, Zhou J, Olson DG, Lynd LR. 2016. A markerless gene deletion and integration system for Thermoanaerobacter ethanolicus. Biotechnol Biofuels 9:100. doi:10.1186/s13068-016-0514-1 27152121 PMC4857275

[38] Zhou J, Shao X, Olson DG, Murphy SJ-L, Tian L, Lynd LR. 2017. Determining the roles of the three alcohol dehydrogenases (AdhA, AdhB and AdhE) in Thermoanaerobacter ethanolicus during ethanol formation. J Ind Microbiol Biotechnol 44:745–757. doi:10.1007/s10295-016-1896-6 28078513

[39] Mougiakos I, Bosma EF, Weenink K, Vossen E, Goijvaerts K, van der Oost J, van Kranenburg R. 2017. Efficient genome editing of a facultative thermophile using mesophilic spCas9. ACS Synth Biol 6:849–861. doi:10.1021/acssynbio.6b00339 28146359 PMC5440800

[40] Jiang Y, Qian F, Yang J, Liu Y, Dong F, Xu C, Sun B, Chen B, Xu X, Li Y, Wang R, Yang S. 2017. CRISPR-Cpf1 assisted genome editing of Corynebacterium glutamicum. Nat Commun 8:15179. doi:10.1038/ncomms15179 28469274 PMC5418603

[41] Liu J, Wang Y, Lu Y, Zheng P, Sun J, Ma Y. 2017. Development of a CRISPR/Cas9 genome editing toolbox for Corynebacterium glutamicum*.* Microb Cell Fact 16:205. doi:10.1186/s12934-017-0815-5 29145843 PMC5693361

[42] Sitara A, Hocq R, Lu AJ, Pflügl S. 2025. Hi-TARGET: a fast, efficient and versatile CRISPR type I-B genome editing tool for the thermophilic acetogen Thermoanaerobacter kivui. Biotechnol Biofuels Bioprod 18:49. doi:10.1186/s13068-025-02647-0 40307869 PMC12044746

[43] Sambrook J, Russell DW, eds. 2001. Molecular cloning: a laboratory manual. Cold Spring Harbor Laboratory Press, Cold Spring Harbor, NY.

