# Supplementary material for: A thermostable Cas9-based genome editing system for thermophilic acetogenic bacterium Thermoanaerobacter kivui
Source: Appl Environ Microbiol. 2025 Sep 8;91(10):e01170-25. doi: 10.1128/aem.01170-25 (PMC12542768; doi:10.1128/aem.01170-25)
Supplement: Supplemental material — Figure S1 and the sequence of plasmid pBlu10-S-P-gH. [file aem.01170-25-s0001.docx]

Supplemental material

**A thermostable Cas9-based genome editing system for thermophilic acetogenic bacterium *Thermoanaerobacter kivui***

Yilin Le^1^, Xue Liu ^1^, Shidong Zhou^1^, Pengju Wu^2^, Mengqi Zhang^2^, Jianzhong Sun*^1^, Jinfeng Ni*^2^, Huilei Wang*^1^

^1^ Biofuels Institute, School of Emergency Management, School of the Environment and Safety Engineering, Jiangsu University, Zhenjiang, Jiangsu 212013, PR China

^2^ State Key Laboratory of Microbial Technology, Shandong University, Qingdao, Shandong, PR China

*Authors for correspondence

(Tel. +86-511-88796122; E-mail: [jzsun1002@ujs.edu.cn](mailto:jzsun1002@ujs.edu.cn); jinfgni@sdu.edu.cn; wanghuilei@ujs.edu.cn)

**Figure S1**

Nucleotide sequences of the 21nt gene-targeting spacer and sgRNA expression cassettes. The tracrRNA and the crRNA were joined by using a GRAA tetraloop to generate a single-guide RNA (sgRNA) as described by Harrington. Blue letters indicate the promoter. Black letters indicate the sgRNA scaffold. Red letters indicate the target sequence of *adh*, and *ldh*, respectively.


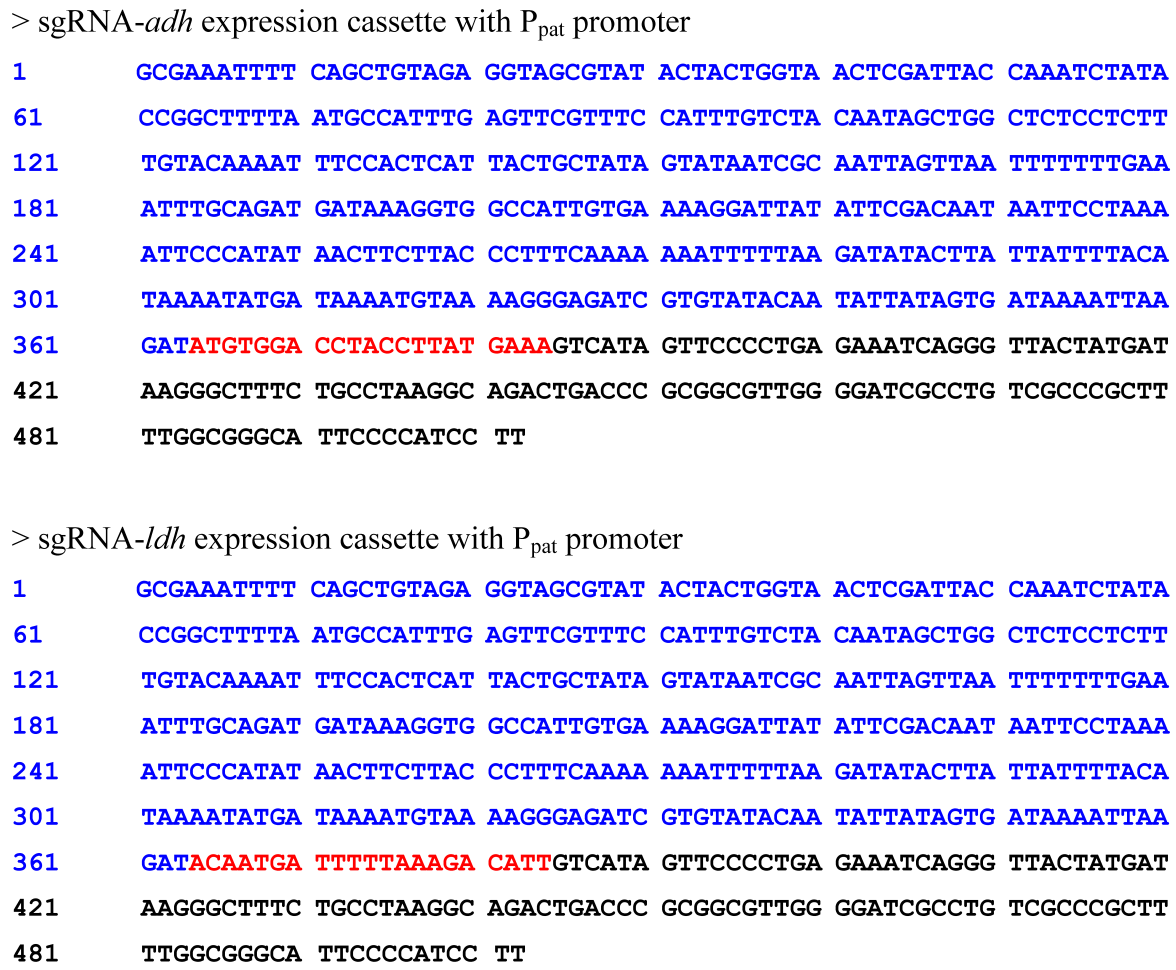


**The sequence of plasmid pBlu10-S-P-gH**

A clear annotation of each element:

130-2494 bp Thermo ori

3048-3810 bp Thermostable kanamycin resistance gene

4151-7414 bp Thermostable Cas9 gene

7857-7995 bp sgRNA

8126-8793 bp pUC ori

8941-9801 bp Ampicillin resistance gene

1 CTAAATTGTA AGCGTTAATA TTTTGTTAAA ATTCGCGTTA AATTTTTGTT AAATCAGCTC

61 ATTTTTTAAC CAATAGGCCG AAATCGGCAA AATCCCTTAT AAATCAAAAG AATAGACCGA

121 GATAGGGTTG AATTGACAAA GTTTTCTATT TGTGTTAACA TTGTTTATAT AATAGTGAAC

181 AGTGTTAAGA TTAAATGTGA GGTGTTTGTA TGGATATTAA TGATTATAAA GAGAAGGGAC

241 TTTATTTATT AAGTAGTATG GATGATTTTA TTAAAATTAA TGATTTGTTT ATGGGTAAAG

301 TTGTTTCTCC TGGCTATGTT GCTTCGGTTT TTGGTGTTTC CAGGTCTACT GTTACACAAT

361 GGATTCAAAG ACGTAAAATT AGAGCTTTTA AGTATAAAGG TAAGGAAGGT GACTATATGG

421 TTATACCTAT TGCTGATATT ATTGATTACA AAAGATTGAG TAATAATGAT TTTATTTATG

481 ATAAGTTAGT GAGGTGATTT ATTTTATGTT TGACGATAGC TATGTTGTTA ATGAGTGTTC

541 GTCTAATGTT AGTGAAAATG ATAGAGATTT TTGTAGTTTG GTTGGTCGTT TTATGATTAT

601 TAATGGTATA GATAAGTTGG TTATTAAGAT TAATAGAAAA TTTAATAGGA AATCTTTAAG

661 TTTAGATTTT AGTGTTGATT TATTCCCTTC TATCAAAGTT TCTGAATTAG TTTTTTTTGA

721 TGAGTTTAAC AAAACGTGTG GTTTTTATTT TTCTTTTAAT TCTTTTACAA TTTTTAAGGC

781 TTTTAGAGAT GTTCATAATC ATAATAAAAT ATCATTTTAT TTTGCATAAT TTCGGGTCTG

841 GGCCGCAGAC CAGGCCCAGT GCTAACAATA TTAATTTTTA ATGTTAGGAA TTGTTTAATT

901 CTTAATTGTG TTTTTAAAGG TAGAATAATT ACCCATTCGC CCTTTAGCCA ACAAAAATTA

961 AGGAGGTATA AACATGGATA AAATGGATTT GATTCTTCAA GATGAAAGAC TGGGTGAGAT

1021 ATTTAAAGAT ATAGATTTAA CAGATAATGA AAAGAGATAT CTTAAATGGT TATGGAAATG

1081 GGATTATGAA ACACGTGATA CTTTTGTATC AATTTTTTTG AAGCTAAAAA ATGGTGGAAA

1141 ATGATTTTTT TCTTATCTTG ATATATTAGA AAAAAGCGTA CTCACGAAGT AAGAATTTGT

1201 AAAAAAAGAA GGGGGGATTT TTTTGGATGA GAGTTTGTAC AAGCAGATTT TAAGTAATAT

1261 TATTATTACT CGTGATTATT GTAAAAATGT TTTAGATAAT ATAAAGTTCA ATGAAAAAAT

1321 AATTGATTAT TATGTTATGT TACAAAATGA TGTTTTTATT GATTTTACTA ATAAAATAAA

1381 TTCAATAAGG GATTGTAATA AATATTGGTA TTTGGATGTT TATAAAAAGC AGAAAATAAA

1441 GGATTTTAAA AAGACTAATT TGTGTAAAGA TAAGTTCTGT AATAATTGTA AGAAAGTTAA

1501 ACAGGCTTCA AGAATGCAAA AATATATTCC TGAATTACAG AAATACAAAG ATGGCTTATA

1561 TCATTTTATA TTTACTGTTG AAAATGTGCC AGGTAGTGAA TTAAGAGATA CTATTGATAG

1621 GTTGTTTAAG TCTTTTAAGT CATTTACAAG GTATTTAAGT GGTAATCTTA AAATAAAAGG

1681 TGTTAATTTT GATAAATGGG GTTATAAAGG CTGTGTAAGG TCTTTAGAGG TAACTTATAG

1741 TATGATTGAT AATCATATTA TGTATCATCC ACACTTGCAT GTTGCGATGA TATTAGATCC

1801 TTTTTACGAT GGTTTTAATG TTGAAAGGAT GCATATAATT AATAAGTTTA GTTATAGCTA

1861 TGGTGTTTTA AAAAGGTTGT TTACTGATGA TGAATTATTA ATTCAAAAAA TTTGGTATTT

1921 ATTGTTTAAT AATATTGAGG TTAACATGGC CAATATAAAT AATTTAGAGG ATGGTTATTC

1981 TTGTTTAGTT AATAAGTTTA GTGATTATGA TTATGCGGAG CTGTTTAAGT ATATTTGTAA

2041 AAATACTGAT GAACAAGGTT TACTTATGAC TTATGATATT TTTAAAGATT TATATTTTGC

2101 ATTACATAAT GTTCATCAGA TACAAGGCTA TGGTTGTTTA TATAATATAA GAGATGATAC

2161 TCAATTAGAT TTAAAGGTTG ATGACATTTA TAATGATTTG ATTGATTTAT TACAAGTTAC

2221 AGAAAATCCT ATACAGTCTA TGGAAACTGT ACAGGATTTA TTAAAGGATA CTGAATATAC

2281 AATAATAAGC CGTAAGCGTA TATTTAAGTA TCTAACACAA TTATATCATA AGGATTGATA

2341 TTTATACCGT CTGTCGGACT CATGCGGAGG GGGACTTGAG GGGGTCTCCC CTCGCATTGT

2401 ACGACAGACG GTATTATTAT TATACAAATT TTTTTTATGT AATTTTTTTT GTGTAATTTT

2461 TTTATACAAA TAATATTTCA ATTCGTCGAC CTGCAGAAGC CTGGCTGCAG GTCGATAAAC

2521 CCAGCGAACC ATTTGAGGTG ATAGGTAAGA TTATACCGAG GTATGAAAAC GAGAATTGGA

2581 CCTTTACAGA ATTACTCTAT GAAGCGCCAT ATTTAAAAAG CTACCAAGAC GAAGAGGATG

2641 AAGAGGATGA GGAGGCAGAT TGCCTTGAAT ATATTGACAA TACTGATAAG ATAATATATC

2701 TTTTATATAG AAGATATCGC CGTATGTAAG GATTTCAGGG GGCAAGGCAT AGGCAGCGCG

2761 CTTATCAATA TATCTATAGA ATGGGCAAAG CATAAAAACT TGCATGGACT AATGCTTGAA

2821 ACCCAGGACA ATAACCTTAT AGCTTGTAAA TTCTATCATA ATTGTGGTTT CAAAATCGGC

2881 TCCGTCGATA CTATGTTATA CGCCAACTTT CAAAACAACT TTGAAAAAGC TGTTTTCTGG

2941 TATTTAAGGT TTTAGAATGC AAGGAACAGT GAATTGGAGT TCGTCTTGTT ATAATTAGCT

3001 TCTTGGGGTA TCTTTAAATA CTGTAGAAAA GAGGAAGGAA ATAATAAATG AAAGGACCAA

3061 TAATAATGAC TAGAGAAGAA AGAATGAAGA TTGTTCATGA AATTAAGGAA CGAATATTGG

3121 ATAAATATGG GGATGATGTT AAGGCAATTG GTGTTTATGG CTCTCTTGGT CGTCAGACTG

3181 ATGGGCCCTA TTCGGATATT GAGATGATGT GTGTTCTGTC AACAGAGGGA GTAGAGTTCA

3241 GCTATGAATG GACAACCGGT GAGTGGAAGG CGGAAGTGAA TTTTTATAGC GAAGAGATTC

3301 TACTAGATTA TGCATCTCGG GTGGAACCGG ATTGGCCGCT TACACATGGT CGATTTTTCT

3361 CTATTTTGCC GATTTATGAT CCAGGTGGAT ACTTTGAGAA AGTGTACCAA ACTGCTAAAT

3421 CGGTAGAAGC CCAAAAGTTC CACGATGCGA TCTGTGCCCT TATCGTAGAA GAGCTGTTTG

3481 AATATGCAGG CAAATGGCGT AATATTCGTG TGCAAGGACC GACAACATTT CTACCATCCT

3541 TGACTGTACA GGTGGCAATG GCAGGTGCCA TGTTGATTGG TCTGCATCAT CGCATCTGTT

3601 ATACGACGAG CGCTTCGGTC TTAACTGAAG CAGTTAAGCA ACCAGATCTT CCTCCAGGTT

3661 ATGTCCAACT GTGCCAGCTC GTAATGTCTG GTCAACTTTC CGACCCTGAG AAACTTCTGG

3721 AATCGCTAGA GAATTTCTGG AATGGGGTTC AGGAGTGGGC GGAACGACAC GGATATATAG

3781 TGGATGTGTC AAAACGCATA CCATTTTGAT GACATTAAGA AAGGTGGTTT TTATGCTCGA

3841 GGATGGATCC ATTTTTGAAT TCGTGAGATA CCCTTGGAAC AGAATGTTTG AAATAGAAGT

3901 TTTACCTAAA ATAATATTAC AACAGGGTTC GTAATGTTAA ATTAATATTA CAGAAATATT

3961 ACAAATTGGT AACATTTATT GACTCATTAA ATACCTGATG CTATAATATA ATCAGGTTGT

4021 TTATCCAAAA CATACTACAT GCTCTAAAAT GTACTTACAT AGGTAAAAAA TTTTTGAAAG

4081 GCATTATGCC TTTCAAAACA AAAAATATAA AAATTAAAAT ACAAATACAA GGAGGAGGAT

4141 TGACTGTGAT **ATGAGATACA AAATCGGCCT TGATATCGGC ATTACCTCTG TAGGTTGGGC**

**4201 AGTCATGAAC TTGGATATTC CCCGCATCGA AGATTTGGGC GTCCGCATTT TTGACAGAGC**

**4261 CGAAAATCCG CAGACGGGAG AATCCCTAGC TCTTCCCCGA CGCCTCGCCC GCTCCGCCCG**

**4321 GCGTCGATTG CGCCGCCGCA AGCATCGGCT GGAGCGCATT CGCCGTCTGG TCATTCGCGA**

**4381 AGGAATTTTA ACGAAAGAGG AACTGGACAA ACTATTCGAG GAAAAACACG AAATCGACGT**

**4441 CTGGCAGCTG CGTGTTGAGG CATTGGATCG AAAATTAAAC AACGACGAGC TGGCTCGCGT**

**4501 CCTCCTTCAT CTAGCCAAGC GGCGCGGCTT CAAATCCAAC CGCAAAAGCG AGCGCAGCAA**

**4561 CAAAGAAAAC AGCACGATGC TCAAACATAT CGAAGAAAAC CGGGCCATTC TCTCAAGCTA**

**4621 CCGGACCGTC GGCGAAATGA TTGTGAAAGA TCCAAAGTTT GCGCTCCATA AGCGCAATAA**

**4681 AGGAGAAAAT TATACAAACA CAATCGCCCG CGATGACTTA GAACGCGAGA TCCGACTCAT**

**4741 TTTCTCCAAA CAGCGTGAGT TTGGAAACAT GAGTTGCACG GAAGAGTTTG AGAATGAATA**

**4801 TATCACCATT TGGGCTTCCC AGCGTCCTGT CGCTTCCAAA GATGACATCG AGAAAAAGGT**

**4861 CGGCTTTTGT ACCTTTGAAC CGAAAGAAAA ACGGGCTCCA AAAGCAACTT ATACATTCCA**

**4921 ATCGTTCATC GCCTGGGAGC ACATCAATAA ATTGCGGCTC ATCTCCCCAT CAGGCGCACG**

**4981 AGGGCTCACC GATGAGGAAC GACGCCTTTT GTATGAACAG GCATTCCAAA AAAACAAAAT**

**5041 CACCTACCAT GATATACGGA CGTTGCTCCA TTTGCCTGAT GACACTTACT TTAAAGGCAT**

**5101 TGTGTATGAT CGAGGCGAAT CACGAAAGCA AAATGAAAAC ATCCGATTCC TTGAACTCGA**

**5161 CGCCTATCAT CAAATTCGGA AAGCCGTCGA TAAAGTGTAT GGAAAAGGGA AGTCAAGTTC**

**5221 ATTTCTCCCA ATCGATTTTG ATACATTCGG TTACGCCCTG ACATTGTTTA AAGATGATGC**

**5281 CGACATTCAC AGCTACTTGC GAAACGAATA TGAACAAAAC GGAAAACGGA TGCCAAATTT**

**5341 AGCGAACAAG GTATATGACA ACGAGCTAAT TGAGGAACTA TTGAACTTGT CATTCACCAA**

**5401 ATTCGGCCAT TTGTCGCTAA AGGCGCTTCG CAGCATTCTC CCGTACATGG AACAAGGAGA**

**5461 AGTCTACTCT TCGGCTTGTG AGCGAGCGGG ATACACATTT ACAGGGCCAA AGAAGAAACA**

**5521 AAAAACAATG TTGCTACCAA ATATTCCGCC GATCGCCAAT CCGGTCGTCA TGCGTGCATT**

**5581 GACACAGGCG CGGAAAGTGG TGAATGCTAT TATTAAAAAG TACGGTTCGC CGGTATCCAT**

**5641 TCATATCGAG TTGGCCCGCG ATTTATCACA AACGTTTGAC GAACGGCGGA AAACAAAAAA**

**5701 AGAACAAGAC GAAAACCGGA AGAAAAACGA AACCGCCATC CGCCAACTCA TGGAGTATGG**

**5761 CTTGACGCTA AACCCAACCG GCCATGACAT TGTCAAGTTC AAACTTTGGA GTGAGCAAAA**

**5821 TGGGAGGTGC GCCTACTCGC TTCAACCGAT CGAAATCGAG CGGCTGCTTG AACCGGGGTA**

**5881 TGTAGAAGTG GATCACGTCA TCCCGTATAG CCGAAGCTTG GACGACAGTT ATACCAATAA**

**5941 AGTGTTGGTA TTGACAAGAG AAAACCGCGA AAAAGGCAAC CGCATTCCTG CCGAATATTT**

**6001 AGGCGTCGGA ACCGAACGCT GGCAACAGTT TGAAACGTTT GTGTTAACGA ACAAGCAGTT**

**6061 TTCCAAAAAG AAACGGGATC GGCTGCTCCG ACTCCATTAT GATGAAAATG AAGAAACAGA**

**6121 ATTTAAAAAT CGAAATTTAA ATGACACCCG GTATATTTCA CGCTTTTTCG CCAACTTTAT**

**6181 TCGCGAACAT CTGAAATTCG CCGAAAGTGA TGACAAGCAA AAAGTCTATA CGGTCAACGG**

**6241 CCGCGTTACC GCCCATTTGC GAAGCCGCTG GGAGTTTAAC AAAAACCGTG AAGAATCGGA**

**6301 TTTGCATCAT GCCGTCGATG CCGCCATCGT CGCTTGCACA ACGCCAAGCG ATATCGCCAA**

**6361 AGTCACCGCC TTTTACCAAC GGCGCGAACA AAACAAAGAA CTGGCCAAAA AGACAGAACC**

**6421 GCACTTCCCG CAGCCTTGGC CGCACTTCGC CGACGAACTG CGGGCGCGTT TATCCAAACA**

**6481 TCCAAAAGAG AGTATAAAAG CTCTCAATCT TGGAAATTAT GATGATCAGA AACTCGAATC**

**6541 GCTACAGCCG GTTTTTGTAT CCCGAATGCC GAAGCGGAGC GTTACAGGGG CGGCTCATCA**

**6601 AGAAACATTA CGGCGCTACG TCGGTATCGA TGAACGGAGC GGGAAAATCC AAACTGTTGT**

**6661 CAAAACGAAA CTGTCCGAAA TCAAGTTGGA TGCGAGCGGG CATTTTCCGA TGTACGGCAA**

**6721 AGAAAGCGAC CCAAGGACAT ACGAAGCGAT CCGCCAGCGT CTGCTTGAAC ATAACAATGA**

**6781 CCCGAAAAAG GCATTCCAAG AACCTTTGTA CAAGCCGAAA AAGAACGGCG AACCCGGGCC**

**6841 TGTCATTCGG ACCGTAAAAA TCATCGACAC AAAAAACCAG GTCATCCCGC TCAATGACGG**

**6901 CAAAACCGTC GCCTACAACA GCAACATCGT TCGGGTTGAC GTATTTGAAA AAGATGGGAA**

**6961 ATATTATTGT GTCCCTGTCT ACACCATGGA CATCATGAAA GGGATTTTGC CAAACAAAGC**

**7021 CATCGAGCCG AACAAGCCGT ACTCTGAGTG GAAGGAAATG ACGGAGGACT ATACGTTCCG**

**7081 ATTTAGCCTG TACCCGAATG ATCTCATTCG CATTGAGCTT CCGCGGGAAA AAACAGTGAA**

**7141 AACAGCCGCA GGCGAAGAGA TCAACGTTAA GGATGTATTT GTCTACTACA AAACGATTGA**

**7201 CTCAGCCAAC GGAGGATTAG AGTTGATCAG CCATGACCAC CGTTTCTCGC TCCGCGGCGT**

**7261 CGGTTCAAGA ACCCTCAAAC GATTCGAGAA ATACCAAGTA GATGTGCTCG GCAACATCTA**

**7321 CAAAGTGAGA GGGGAAAAAA GAGTTGGGTT GGCGTCATCT GCTCATTCGA AAACCGGGGA**

**7381 AACTGTCCGT CCGTTACAAT CAACTCGTGA TTGA**TAGTAC CTAGATTTAG ATGTCTAAAA

7441 AGCTTTTTAG ACATCTAATC TTTTCTGAAG TACATCCGCA ACTGTCCATA CTCGCGAAAT

7501 TTTCAGCTGT AGAGGTAGCG TATACTACTG GTAACTCGAT TACCAAATCT ATACCGGCTT

7561 TTAATGCCAT TTGAGTTCGT TTCCATTTGT CTACAATAGC TGGCTCTCCT CTTTGTACAA

7621 AATTTCCACT CATTACTGCT ATAGTATAAT CGCAATTAGT TAATTTTTTT GAAATTTGCA

7681 GATGATAAAG GTGGCCATTG TGAAAAGGAT TATATTCGAC AATAATTCCT AAAATTCCCA

7741 TATAACTTCT TACCCTTTCA AAAAAATTTT TAAGATATAC TTATTATTTT ACATAAAATA

7801 TGATAAAATG TAAAAGGGAG ATCGTGTATA CAATATTATA GTGATAAAAT TAAGAT**ATGT**

**7861 GGACCTACCT TATGAAAGTC ATAGTTCCCC TGAGAAATCA GGGTTACTAT GATAAGGGCT**

**7921 TTCTGCCTAA GGCAGACTGA CCCGCGGCGT TGGGGATCGC CTGTCGCCCG CTTTTGGCGG**

**7981 GCATTCCCCA TCCTT**AACGA AGACCTTGGC GGAAAACGTC AAGGTCTTTT TTTCTAGATA

8041 GTACCTAGAT TTAGATGTCT AAAAAGCTTT TTAGACATCT AATCTTTTCT GAAGTACATC

8101 CGCAACTGTC CATACTCAGA ACATGTGAGC AAAAGGCCAG CAAAAGGCCA GGAACCGTAA

8161 AAAGGCCGCG TTGCTGGCGT TTTTCCATAG GCTCCGCCCC CCTGACGAGC ATCACAAAAA

8221 TCGACGCTCA AGTCAGAGGT GGCGAAACCC GACAGGACTA TAAAGATACC AGGCGTTTCC

8281 CCCTGGAAGC TCCCTCGTGC GCTCTCCTGT TCCGACCCTG CCGCTTACCG GATACCTGTC

8341 CGCCTTTCTC CCTTCGGGAA GCGTGGCGCT TTCTCATAGC TCACGCTGTA GGTATCTCAG

8401 TTCGGTGTAG GTCGTTCGCT CCAAGCTGGG CTGTGTGCAC GAACCCCCCG TTCAGCCCGA

8461 CCGCTGCGCC TTATCCGGTA ACTATCGTCT TGAGTCCAAC CCGGTAAGAC ACGACTTATC

8521 GCCACTGGCA GCAGCCACTG GTAACAGGAT TAGCAGAGCG AGGTATGTAG GCGGTGCTAC

8581 AGAGTTCTTG AAGTGGTGGC CTAACTACGG CTACACTAGA AGGACAGTAT TTGGTATCTG

8641 CGCTCTGCTG AAGCCAGTTA CCTTCGGAAA AAGAGTTGGT AGCTCTTGAT CCGGCAAACA

8701 AACCACCGCT GGTAGCGGTG GTTTTTTTGT TTGCAAGCAG CAGATTACGC GCAGAAAAAA

8761 AGGATCTCAA GAAGATCCTT TGATCTTTTC TACGGGGTCT GACGCTCAGT GGAACGAAAA

8821 CTCACGTTAA GGGATTTTGG TCATGAGATT ATCAAAAAGG ATCTTCACCT AGATCCTTTT

8881 AAATTAAAAA TGAAGTTTTA AATCAATCTA AAGTATATAT GAGTAAACTT GGTCTGACAG

8941 TTACCAATGC TTAATCAGTG AGGCACCTAT CTCAGCGATC TGTCTATTTC GTTCATCCAT

9001 AGTTGCCTGA CTCCCCGTCG TGTAGATAAC TACGATACGG GAGGGCTTAC CATCTGGCCC

9061 CAGTGCTGCA ATGATACCGC GAGACCCACG CTCACCGGCT CCAGATTTAT CAGCAATAAA

9121 CCAGCCAGCC GGAAGGGCCG AGCGCAGAAG TGGTCCTGCA ACTTTATCCG CCTCCATCCA

9181 GTCTATTAAT TGTTGCCGGG AAGCTAGAGT AAGTAGTTCG CCAGTTAATA GTTTGCGCAA

9241 CGTTGTTGCC ATTGCTACAG GCATCGTGGT GTCACGCTCG TCGTTTGGTA TGGCTTCATT

9301 CAGCTCCGGT TCCCAACGAT CAAGGCGAGT TACATGATCC CCCATGTTGT GCAAAAAAGC

9361 GGTTAGCTCC TTCGGTCCTC CGATCGTTGT CAGAAGTAAG TTGGCCGCAG TGTTATCACT

9421 CATGGTTATG GCAGCACTGC ATAATTCTCT TACTGTCATG CCATCCGTAA GATGCTTTTC

9481 TGTGACTGGT GAGTACTCAA CCAAGTCATT CTGAGAATAG TGTATGCGGC GACCGAGTTG

9541 CTCTTGCCCG GCGTCAATAC GGGATAATAC CGCGCCACAT AGCAGAACTT TAAAAGTGCT

9601 CATCATTGGA AAACGTTCTT CGGGGCGAAA ACTCTCAAGG ATCTTACCGC TGTTGAGATC

9661 CAGTTCGATG TAACCCACTC GTGCACCCAA CTGATCTTCA GCATCTTTTA CTTTCACCAG

9721 CGTTTCTGGG TGAGCAAAAA CAGGAAGGCA AAATGCCGCA AAAAAGGGAA TAAGGGCGAC

9781 ACGGAAATGT TGAATACTCA TACTCTTCCT TTTTCAATAT TATTGAAGCA TTTATCAGGG

9841 TTATTGTCTC ATGAGCGGAT ACATATTTGA ATGTATTTAG AAAAATAAAC AAATAGGGGT

9901 TCCGCGCACA TTTCCCCGAA AAGTGCCAC
